# Network reconstruction and validation of the Snf1/AMPK pathway in baker’s yeast based on a comprehensive literature review

**DOI:** 10.1038/npjsba.2015.7

**Published:** 2015-10-22

**Authors:** Timo Lubitz, Niek Welkenhuysen, Sviatlana Shashkova, Loubna Bendrioua, Stefan Hohmann, Edda Klipp, Marcus Krantz

**Affiliations:** 1 Theoretical Biophysics, Humboldt-Universität zu Berlin, Berlin, Germany; 2 Department of Chemistry and Molecular Biology, University of Gothenburg, Göteborg, Sweden

## Abstract

**Background/Objectives:**

The SNF1/AMPK protein kinase has a central role in energy homeostasis in eukaryotic cells. It is activated by energy depletion and stimulates processes leading to the production of ATP while it downregulates ATP-consuming processes. The yeast SNF1 complex is best known for its role in glucose derepression.

**Methods:**

We performed a network reconstruction of the Snf1 pathway based on a comprehensive literature review. The network was formalised in the rxncon language, and we used the rxncon toolbox for model validation and gap filling.

**Results:**

We present a machine-readable network definition that summarises the mechanistic knowledge of the Snf1 pathway. Furthermore, we used the known input/output relationships in the network to identify and fill gaps in the information transfer through the pathway, to produce a functional network model. Finally, we convert the functional network model into a rule-based model as a proof-of-principle.

**Conclusions:**

The workflow presented here enables large scale reconstruction, validation and gap filling of signal transduction networks. It is analogous to but distinct from that established for metabolic networks. We demonstrate the workflow capabilities, and the direct link between the reconstruction and dynamic modelling, with the Snf1 network. This network is a distillation of the knowledge from all previous publications on the Snf1/AMPK pathway. The network is a knowledge resource for modellers and experimentalists alike, and a template for similar efforts in higher eukaryotes. Finally, we envisage the workflow as an instrumental tool for reconstruction of large signalling networks across Eukaryota.

## Introduction

The adenosine monophosphate-activated protein kinase (AMPK) is the key regulator of energy homeostasis in eukaryotic cells. It orchestrates cellular adaptation to nutrient availability and ensures cell survival under stressful conditions.^1^ Its central role in energy regulation makes it a potential drug target for treating diseases like obesity, heart diseases or type 2 diabetes. Thus, the mechanisms of activation of and signalling through AMPK have received great attention.

The Sucrose Non-Fermenting kinase Snf1 is the AMPK orthologue in baker’s yeast, *Saccharomyces cerevisiae*.^2^ Yeast cells adjust their metabolism in response to nutrient availability to ensure cell survival.^3^ Glucose and fructose cause the repression of numerous genes encoding nutrient uptake systems and metabolic enzymes, and cells shifted to less preferred carbon sources change their transcriptome and proteome to utilise new carbon sources.^4^ This reprogramming is mediated by the Snf1 kinase, which is a key regulator of energy metabolism also in yeast.^5^

The architecture of the Snf1 pathway is well-known. At the core of the Snf1 pathway is the heterotrimeric SNF1 complex, consisting of a catalytic α-subunit (Snf1), a regulatory γ-subunit (Snf4) and one of three β-subunits (Sip1, Sip2 and Gal83) that serve as scaffold and targeting subunits. These three forms of the SNF1 complex have overlapping but distinct roles and localisation patterns, and are regulated by upstream kinases, phosphatases and other modifiers. SNF1 in turn regulates a range of cytoplasmic and nuclear targets, in particular the transcription factors responsible for the reprogramming of energy metabolism. Hence, also the physiological role of the pathway is well-defined.

These features make the Snf1 system a suitable target for network reconstruction. This process is well-established for metabolic networks, where a high degree of conservation enables sequence-based reconstruction.^6^ However, the specificities of signalling components are encoded in short or ill-defined sequences, and may be completely disrupted by point mutations.^7^ Therefore, reconstruction of signalling networks relies on experimentally validated reactions as extracted from literature, and the validation on physiological input/output relationships. Various approaches have been used to generate large, primarily graphical, signalling network reconstructions.^8–11^ However, most of these cannot be simulated and validated, as routinely done for metabolic networks.^12^ Thus, we need an integrated workflow for reconstruction, validation and refinement of signalling networks.

Here, we present such a workflow and apply it to create a reconstruction of the Snf1 network. We performed an exhaustive literature review with the explicit aim to collect all mechanistic information on the signal transfer, and to compile that information in a stringent, machine-readable format. We chose the rxncon language for its scalability and fidelity to the empirical data structure,^11,13^ and used the rxncon toolbox for model creation, validation and gap filling.^14^ We extended the literature-curated network to enable information transfer between each input and every output that it triggers. The resulting network fully reproduces the expected qualitative behaviour in Boolean simulations, and each of the gap filling additions constitutes a clearly phrased hypothesis awaiting experimental validation. Finally, we generated a rule-based model corresponding to the final reconstruction as a proof-of-principle. We provide both the initial literature curation and the gap-filled network model as community resources that summarise the complete Snf1 literature to date. We envisage them as useful tools in yeast Snf1/AMPK research and as templates for similar efforts in higher organisms, and the workflow as a key tool for large scale reconstruction of signalling networks.

## Materials and Methods

As described in detail in the Supplementary Material section (supplementary information 1), the reconstruction was performed using the rxncon language and tool.^11^ During the reconstruction process, we collected two kinds of data from literature: mechanistic and physiological/functional data. The mechanistic data were further divided into elemental reactions and contingencies. The elemental reactions define possible state transition events that produce or consume elemental states. Importantly, the elemental states define only a single property of a component, such as a specific modification or binding. Hence, they correspond to the full set of specific states for which that modification and binding is true, and, correspondingly, an elemental reaction corresponds to a set of reactions (reviewed in ref. 13). These decontextualised reactions are equivalent to the protein–protein interactions in e.g., the BioGRID database.^15^ The contingency information defines how elemental reactions depend on elemental states, and hence defines the causality in the network. The distinction between reactions and contingencies is the same as in the SBGN entity relationship diagrams,^16^ and together the reactions and contingencies fully define the network and can be used for automatic model generation (Supplementary file 1; ref. 14).

The physiological/functional data were used for validation of the network reconstruction. We searched for inputs known to activate Snf1 and for the downstream Snf1-dependent responses to these inputs, which we collected as a set of input/output relationships. For validation, we generated and simulated the corresponding bipartite Boolean model (bBM) with the rxncon toolbox, and visualised the attractor states on the regulatory graph in Cytoscape.^14,17^ We analysed only the attractor states, which are the end results of the simulations, due to the very crude time concept in Boolean models. The attractor states correspond to a qualitative steady state, which can be used to determine if the signal is transduced through the network or not. We scored functionality for each input–output relationship by determining if that output responded appropriately when the input was changed between on and off (Table 1). When necessary, we adapted the network definition to resolve blocks and/or constitutive activities as detailed in the Results section. All such adaptations have been clearly labelled as hypotheses in the updated network definition (Supplementary file 2). Finally, we translated the gap-filled network into a rule-based model in the BioNetGen language.^18^ All methods are described in more detail in the Supplementary Methods section.

## Results

### The Snf1 network reconstruction is based on a comprehensive literature review

We present a network reconstruction of the Snf1 pathway based on a comprehensive literature review. We used the Textpresso tool at *Saccharomyces* Genome Database (SGD) to search the literature with the ‘Snf1’ string, and extended this list with further papers manually. In total, we could find and download 444 publications dating from February 1977 to January 2015 from online research literature repositories (Supplementary file 3). We read and evaluated each of these papers, searching for and re-evaluating experimental evidence of interactions of pathway components. We extracted mechanistic information on the Snf1 pathway from 77 papers.^19–95^ The literature-derived network reconstruction (NR1) encompasses 71 reactions and 105 contingencies, each of which is associated with ⩾1 references (Supplementary file 1). Hence, this initial network reconstruction is fully referenced and based on careful manual curation of the entire Snf1 literature.

### The network reconstruction encompasses 52 components taking part in 71 elemental reactions

**Figure 1:**
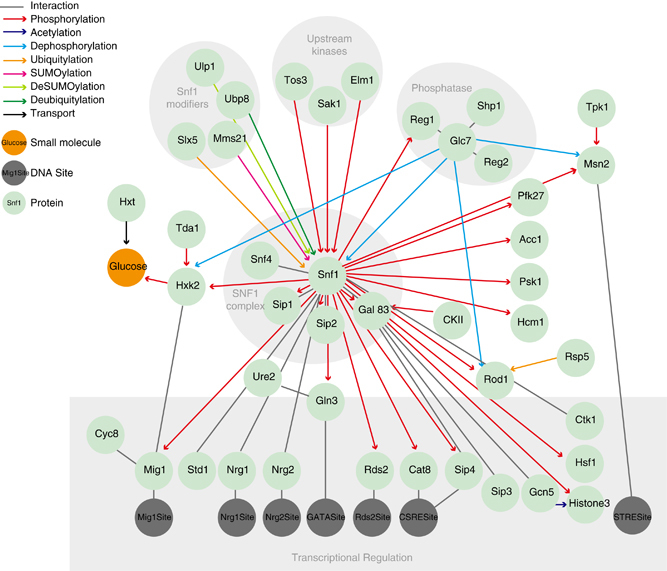
The Snf1 pathway and its components. The reaction graph depicts the pathway components of the NR1 network, and the different reactions they take part in (excluding the nuclear import and export reactions, and the nuclear pore complex mediating them). The SNF1 complex is regulated by kinases, phosphatases and other modifiers shown at the top. The transcriptional regulation is shown at the bottom. The coloured circles represent components and coloured edges represent different classes of reactions. The information in the reaction graph is only topological, and the edges cannot be interpreted in terms of information flow. However, most of them do carry information, as shown in the regulatory graph (Figure 2).

At the topological level, NR1 encompasses 52 components and 71 elemental reactions (Figure 1). The components are proteins (44), small molecules (1) and transcription factor-binding sites (7). The signalling pathway displays a clear bow-tie structure centred on the SNF1 kinase complex, which participates in 40 of the 71 reactions. The activity of the SNF1 kinase complex is controlled by posttranslational modification by a battery of upstream regulators. The key modification is phosphorylation of Thr210 in Snf1, which is indispensable for kinase activity. This residue is phosphorylated by any of the three upstream kinases Sak1, Elm1 or Tos3,^75^ and dephosphorylated by phosphoprotein phosphatase type 1 (PP1); consisting of the catalytic subunit Glc7 and either of the regulatory subunits Reg1 and Reg2.^75^ In addition, the SNF1 complex is regulated by ubiquitylation and sumoylation of the catalytic Snf1 subunit. The active SNF1 complex in turn regulates a wide range of targets that are primarily involved in energy metabolism and transcription.

### The signal transmission in the Snf1 pathway is well-understood

**Figure 2:**
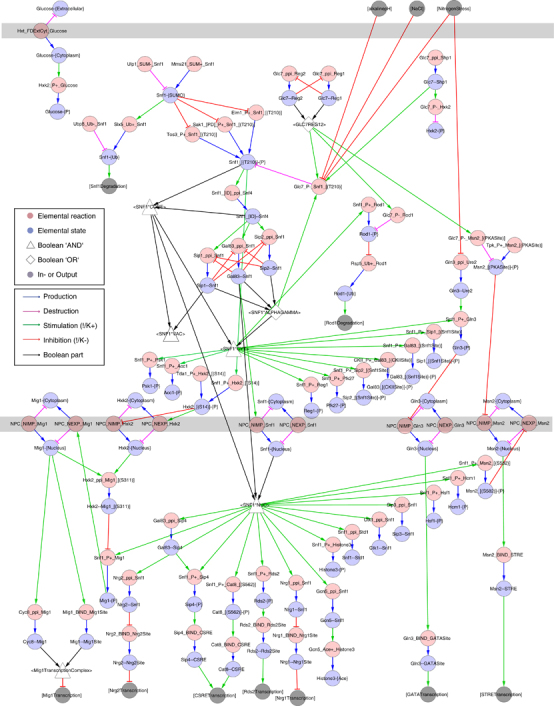
The regulatory structure of the Snf1 pathway. The regulatory graph depicts the information flow through the NR1 network as a bipartite-directed graph. Elemental reactions (red nodes) produce or consume elemental states (blue nodes) via reaction edges, which correspond to the edges in the reaction graph (Figure 1). The elemental states in turn influence the elemental reactions via contingency edges, and they define contextual constraints on reactions. The inputs in form of external stimuli can be found at the top of the picture, as grey nodes with node names in square brackets. The cytoplasmic reactions and SNF1 complex formation are found in the middle part, and the nuclear reactions at the bottom with the transcriptional output again as grey nodes with names in square brackets. Information passes through the network along the unidirectional edges, either by production (blue edges) or consumption (purple edges) of elemental states by reactions, or by the regulatory effect of elemental states on elemental reactions. Positive contingencies from activating states to activated reactions are denoted in green, while inhibitory contingencies appear in red. More complex requirements, such as formation of the active forms of the SNF1 complex, are defined by Boolean states that are indicated as white triangles (AND) or diamonds (OR). The compartment borders in grey represent the plasma membrane and the nuclear membrane, and have been included as visual guides but carry no information. At the center, we have the three active forms of the SNF1 complex; SNF1*CYT, SNF1*VAC and SNF1*NUC, that are localised to the cytoplasm, the vacuole and the nucleus, respectively. Information can only pass along the direction of the edges, and we can follow them from inputs to outputs. In this network we have four inputs: The three grey stress inputs to the upper right, and extracellular glucose to the upper left. The last is part of an unconnected subgraph, highlighting the fact that we do not know how the glucose signal is sensed by the pathway. The original NR1 Cytoscape file is attached as Supplementary file 5.

At the regulatory level, NR1 encompasses 71 elemental reactions that produce or consume 64 elemental states, which in turn regulate the reactions via 108 contingencies (Figure 2; ref. 11). The network retains the bow-tie structure also at the regulatory level, where it is centred on the active forms of the SNF1 complex. The graph is well-connected, meaning that the causal relationships between reactions and states are well-known. Importantly, there are directed paths from the inputs to the outputs, indicating that the mechanism of information transfer is understood at the molecular, mechanistic level. However, there is one disconnected subgraph, reflecting our lack of knowledge on how—at a mechanistic level—glucose and energy regulate the Snf1 pathway.

### Input/output validation reveals specific knowledge gaps

To validate NR1, we made use of the well-understood input/output relationship of the Snf1 pathway. We examined whether the network reconstruction sufficed to enable information transfer through the network as expected. For this purpose, we used a bBM to determine if the attractor states for every input combination correspond to that expected based on our empirical understanding of the pathway. Examining the input/output relationship of the Snf1 network reconstruction, we found that none of the documented input/output relationships could be reproduced by the bBM of NR1 (Table 1), highlighting important mechanistic gaps in the combined literature knowledge.

### The gap filling process required three steps to generate a functional network model

**Figure 3:**
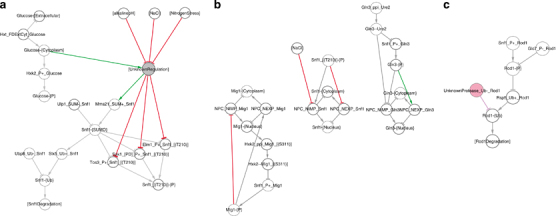
Three gap filling steps suffice to fully connect inputs with their respective outputs. The figure displays the difference between the initial (NR1) and final (NR2) networks. (**a**) Connection of the glucose subgraph to the main pathway. This connection required one abstract state, which is active in the presence of glucose as long as none of the stresses are active, and which activates sumoylation and inhibits phosphorylation of Snf1. It also activates the phosphorylation of Msn2. (**b**) Adaptation of transport reactions. The nuclear localisation pattern could only be reproduced if both import and export are regulated, otherwise the localisation oscillates. In addition, we added a direct edge from salt stress to inhibit Snf1 nuclear localisation, to account for the observation that Snf1 is phosphorylated but not nuclear on salt stress. (**c**) Deubiquitylation of Rod1. An unknown deubiqitylating enzyme was added, acting on Rod1. Faded nodes and edges were part of the initial network reconstruction (NR1), while nodes and edges with full colours were added to NR2 during the network refinement. The gap filled NR2 Cytoscape file is attached as Supplementary file 6.

**Figure 4:**
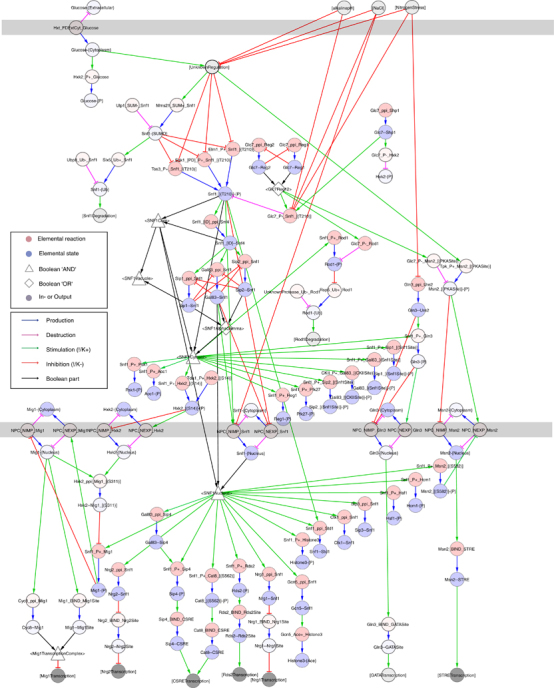
The steady state of the Snf1 bBM under glucose deprivation is a point attractor. The end state of the bBM simulation under glucose-deprived conditions visualised on the regulatory graph of the updated network. The bBM was generated automatically based on the final network after validation, and the simulation initiated from the starting states defined in the Methods section. The pale nodes are inactive and the filled nodes are active. The Boolean model is attached in the BooleanNet and BoolNet formats as Supplementary files 7 and 8, respectively.

We used iterative network improvement, bBM generation and validation as described above to identify and fill the gaps in the network. We identified blocks in the information transfer, i.e., reactions or states that do not vary as expected in response to varying inputs, and eliminated these blocks by minimal modifications of the network (Figure 3). The gap filling workflow on the Snf1 network reconstruction identified a total of 1 missing component, 1 missing reaction and 13 missing contingencies (Table 2), and resulted in a final network (NR2) with 53 components, 72 reactions and 118 contingencies (Supplementary file 2). The corresponding bBM reproduced the expected input/output relationships in all tested cases, as shown for glucose deprivation (Figure 4), basic conditions (Supplementary file 9), salt stress (Supplementary file 10), alkaline conditions (Supplementary file 11) and nitrogen starvation (Supplementary file 12). NR2 is a merge of the explicitly referenced original curation and clearly labelled hypothetical reactions and contingencies, each of which correspond to a testable hypothesis.

### The rxncon network definition corresponds to a dynamic model

As proof-of-principle, we used the rxncon toolbox and NR2 to generate a rule-based model in the BioNetGen language (Supplementary file 4). The resulting model has 176 distinct parameters, precluding reliable parameter estimation based on current data and hence meaningful analysis. However, the model can be simulated using NFsim,^96^ demonstrating that the rxncon network reconstruction can be used as basis for dynamic simulations.

## Discussion

**Figure 5:**
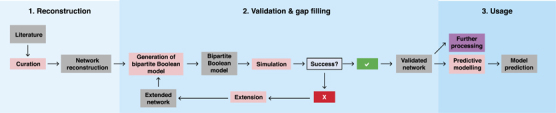
The network reconstruction workflow. The first phase of the process was the network reconstruction itself, where we extracted reaction and contingency information from published data. The curation process produced the initial network reconstruction (NR1). The second phase of the process was the validation and iterative improvement. It starts with the creation of the bBM, followed by simulation of the bBM and comparison to the known input/output relationships. If the model fails to transmit information as expected, the gap is identified and filled, and the updated network re-enters the workflow at model creation. If the model passes all tests, it is accepted and considered a validated network (NR2). The validated network constitutes a knowledge resource, but can also be further processed with the rxncon tool to generate graphical or mathematical models in a range of formats, as illustrated by the rule-based model (Supplementary file 4).

Here, we present a network reconstruction of the Snf1 signalling pathway. Network reconstruction is well-established for metabolic networks, and they are divided into four stages: (i) draft reconstruction, (ii) refinement, (iii) conversion into a computational model and (iv) network evaluation (including gap filling).^12^ However, these methods cannot directly be applied to signal transduction networks, and hence we developed an analogous, but distinct, workflow (Figure 5).

The first and arguably most important phase is the translation of diverse experimental findings into a single, machine-readable reconstruction of the system under study. For this purpose, we carefully curated and distilled the complete literature on the Snf1/AMPK pathway architecture into a network reconstruction. We searched for evidence of reactions between network components and of causal relationships between reactions (i.e. contingencies), and formalised the reactions and contingencies in the rxncon language. The resulting network is fully annotated, machine readable and can be used for automatic model generation with the rxncon software tool. Importantly, we found text mining insufficient for high quality network reconstruction and based the curation on manual re-evaluation of the data presented in the cited papers. Hence, this first phase corresponds to the first and second stages of metabolic network reconstruction.^12^ The result is a high-quality curation summarising the entire Snf1 literature in a fully annotated, machine-readable format.

The second phase is the network validation. This is again well-established for metabolic networks, based on the assumption that mass transfer paths exist such that all metabolites can be reached from the input nutrients.^97^ Again, this does not hold here, as there is no mass transfer through a signalling network. Instead, we used the known input/output relationships to define information paths through the network. The first step of phase II is the generation of a computational model, corresponding to stage 3 of metabolic network reconstruction.^12^ Here, we used the rxncon tool to generate and simulate the bBM corresponding to the network reconstruction, and used the bBM to determine whether information paths are functional.^14^ This analysis revealed that none of the input/output paths could be reproduced based on the literature curation itself (Table 1). Thus, critical information was missing from our understanding of the Snf1 pathway.

To identify these missing links, we went through an iterative gap filling and validation process. The bBM was modelled with alternating inputs to determine which paths worked as expected. For those that did not, we identified the missing steps manually and modified the network to solve the problem. After modification, a new bBM was generated and evaluated, and the process repeated until all paths were functional (Table 1). The modifications fall into one of three classes: missing causal links (i.e. contingencies), states that could not be consumed (missing reverse reactions) or artefacts from the binary assumptions in the Boolean model (missing/modified contingencies). Each modification corresponds to a testable hypothesis, and is clearly labelled as such to distinguish it from the reactions and contingencies that are based on existing literature evidence (Supplementary file 2). The result is a validated, functional network that qualitatively reproduces the input/output paths known form empirical observations.

The gap filling modifications fall into three groups (Figure 3; Table 2). First, the reconstruction lacks a connection between intracellular glucose and phosphorylation of Snf1, consistent with the current (lack of) understanding of how the pathway is activated. To enable the response to glucose, we included an unknown regulatory step connecting glucose and stress signals to Snf1, as proposed in ref. 98. It stimulates Snf1 sumoylation and inhibits phosphorylation such that Snf1 is activated on glucose deprivation. Second, we adapted the trans-organelle transport reactions. Both import and export need to be regulated to prevent components from cycling between compartments. While this is an artefact from the binary assumptions in the bBM, it is also likely that the nucleo-cytoplasmic shuttling is regulated in both directions to avoid futile cycling *in vivo*. Third, we added consumption reactions for states lacking them. Here, Rod1 is ubiquitylated by Rsp5 but the reverse reaction is unknown. To avoid permanent ubiquitylation of Rod1, we introduced an unknown deubiquitylating peptidase. Twenty such enzymes exist in yeast, although activity against Rod1 has not been reported.^99^ Hence, the gaps identified in the validation process could be filled with reactions and contingencies that are consistent with our knowledge, and which constitute explicit testable hypotheses.

The mechanism of activation remains an open question. Snf1 responds to a number of stress factors in addition to glucose limitation, namely high salt concentrations, alkaline pH and nitrogen limitation.^44,100^ The proposal that energy is the main cue is consistent with the wide but specific range of stresses that induces the pathway, and the unknown regulatory step we included may turn out to be a direct measure of the energy state of the cell. The strongest candidate is nucleotide binding, consistent with how AMPK works in higher organisms. There is some evidence in this direction, as ADP protects Snf1 from dephosphorylation^101^ and AMP appears to interact with Snf4.^102^ However, there are also reports that the SAGA acetyl transferase complex deubiquitylates Snf1, thereby affecting Snf1 kinase activity, Snf1 phosphorylation and SNF1 complex stability.^103^ Ubiquitylation is stimulated by SUMOylation, and Snf1 is SUMOylated by the SUMO-E3 ligase Mms21 under glucose conditions.^93^ As a consequence, Snf1 is ubiquitylated by Slx5-Slx8 and degraded.^104^ Thus, the mechanism remains elusive, and we summarise this gap in a single, glucose-regulated step in the final network.

To create this network reconstruction, we introduced a workflow for network reconstruction, validation and iterative refinement. Similar workflows have proven crucial for the success in large scale metabolic network reconstruction and modelling, but have hitherto not been available for signalling networks. Here, we demonstrate that this workflow works for a well-defined pathway. However, the challenges will aggravate as we extend the scope toward larger and eventually genome scale networks. First, the knowledge gaps will be much more severe in the grey areas between the traditional pathways as compared with their well-studied cores. This information bias is likely one of the reasons for the clear bow-tie structures in the SNF network (Figures 1 and 2). Second, the demands on the formats and methods increase drastically with larger network size. Several large mapping efforts have used the specific state based process description format, and these maps are highly valued community resources.^8–10^ Here, we chose a reaction-contingency based format, as the network definition scales more favourably with network size and has better congruence with experimental data (reviewed in ref. 13). This choice also enabled the use of the rxncon toolbox with the automatic model generation that was necessary for the iterative validation and gap filling processes. As we illustrate with the rule-based model generation, the rxncon network definition can also be used to create a dynamic model. However, meaningful parameterisation and efficient simulation of such large models are still outstanding challenges. Finally, the rxncon language enables clean bottom-up reconstructions, as each reaction and contingency can be defined independently of other reactions and contingencies, respectively. This stands in stark contrast to the specific state-based formats, where reactions and causalities are weaved together and must be adapted as new information appears. Taken together, the proposed workflow provides an approach to tackle large networks with partially very sparse knowledge. We are convinced that this or a similar approach will be instrumental in the reconstructions of genome scale signalling networks in eukaryotes.

## Figures and Tables

**Table 1:** The input/output relationships in the Snf1 pathway

*Input*	*Output*	*Recon 0*	*Recon I*	*Recon II*	*Recon III*	*Expected behaviour*	*Reference PMID*
Glucose−	[Snf1Degradation]	Fail	Pass	Pass	Pass	OFF	21628526
	[Rod1Degradation]	Fail	Fail	Fail	Pass	OFF	22249293
	[Mig1Transcription]	Fail	Fail	Pass	Pass	ON	2167835, 14871952, 2002006, 1541392, 17178716
	[Nrg1Transcription]	Fail	Fail	Pass	Pass	ON	12024013
	[Nrg2Transcription]	Fail	Fail	Pass	Pass	ON	12024013
	[CSRETranscription]	Fail	Fail	Pass	Pass	ON	9111319, 15121831
	[Rds2Transcription]	Fail	Fail	Pass	Pass	ON	17875938
	[STRETranscription]	Fail	Pass	Pass	Pass	ON	12093809
							
pH 8	[Mig1Transcription]	Fail	Fail	Pass	Pass	ON	22372618
	[Nrg1Transcription]	Fail	Fail	Pass	Pass	ON	12509465, 17023428
	[STRETranscription]	Fail	Pass	Pass	Pass	ON	21749328
							
Nitrogen−	[GATATranscription]	Fail	Fail	Pass	Pass	ON	11809814, 12062797
							
NaCl+	[STRETranscription]	Fail	Pass	Pass	Pass	ON	8641288

For each input condition, the table lists the expected state of each outputs, as well as the observed output in the simulation of (0) the initial network reconstruction (NR1), (1) after the first round of refinement, (2) after the second round of refinement and (3) in the final model (NR2). The PMIDs indicate references to experimental observations of these input/output relationships. The reference condition is the presence of glucose and the absence of any stress (Supplementary File 9), and entries are only given when they have been reported to differ from the reference condition.

**Table 2:** The three gap-filling steps

*Reconstruction*	*Modification*
Recon I	Unknown glucose-regulated step (US): stimulated by intracellular glucose, inhibited by NaCl, pH8, nitrogen limitation
	If US active:
	SUMOylation of Snf1 is stimulated, deSUMOylation inhibited
	Elm1, Sak1, Tos3 are inhibited
	phosphorylation of Msn2 by Tpk1 is stimulated
	
Recon II	phosphorylated Mig1 cannot be imported into the nucleus
	phosphorylated Snf1 cannot be exported from the nucleus
	NaCl prevents Snf1 import to nucleus
	phosphorylation of Gln3 induces nuclear export
	
Recon III	Rsp5 antagonizing protease for Rod1

The table indicates the modifications done in each of the three refinement steps.
