# Supplementary material for: Network reconstruction and validation of the Snf1/AMPK pathway in baker’s yeast based on a comprehensive literature review
Source: NPJ Syst Biol Appl. 2015 Oct 22;1:15007–. doi: 10.1038/npjsba.2015.7 (PMC5516868; doi:10.1038/npjsba.2015.7)
Supplement: Supplementary methods [file npjsba20157-s1.pdf]

Supplementary methods

The rxncon language

The network reconstruction was performed using the reaction-contingency (rxncon) language (1). The language is based on the separation of two distinct classes of information: 1) *Elemental reactions* define the different events that can occur within the network and 2) *Contingencies* define the causal relationship between the reactions. The same distinction is used in e.g. the Entity Relationship diagrams of the Systems Biology Graphical Notation (SBGN-ER; (2)). Elemental reactions produce or consume *elemental states*, such as a specific protein-protein interaction or the phosphorylation of a specific protein residue. Importantly, they do not define any other aspect of the reactants, meaning that they correspond to sets of specific state transitions. Consequently, the elemental states correspond to sets of specific states. The contingencies define the causal relationships, i.e. how elemental reactions depend on elemental states, and thus how previous reactions stimulate or inhibit downstream events. Taken together, the reaction and contingency information fully define the network.

The elemental reactions are defined in the rxncon reaction list as “subject-verb-object” clauses, which are equivalent to the format used to document protein-protein interactions in e.g. the BioGRID database (3). In the case of transport or catalytic modification, the channel or enzyme takes the subject position. For reciprocal reactions such as protein-protein interactions, the components are assigned as subject or object based on alphabetic order, to keep reaction names deterministic. In the Snf1 network reconstruction, we used the following twelve reactions:

Ace+	acetylation
BIND	DNA binding
FDExtCyt	facilitated diffusion: External to Cytoplasm
NEXP	nuclear export
NIMP	nuclear import
P+	phosphorylation
P-	dephosphorylation
ppi	protein-protein interaction
SUM+	sumoylation
SUM-	desumoylation
Ub+	ubiquitylation
Ub-	deubiquitylation

The contingencies are defined in the rxncon contingency list as “subject-verb-agent” passive clauses. They take three distinct subjects: reactions, outputs and Booleans. The Boolean contingencies define more complex requirements as AND or OR combinations of elemental states, inputs or other Boolean states. The reaction contingencies define the effect of an elemental state, an input or a Boolean state on an elemental reaction. Finally, output contingencies are like reaction contingencies, but target an output. Inputs and outputs define the network boundaries and are the interface with everything not included in the model. [Inputs] and [Outputs] appear in square brackets, while <Booleans> appear in pointy brackets. The contingency list allows the following operators:

	Contingency	
Contingencies:	!	Absolutely required
	K+	Positive effector
	0	No effect
	K-	Negative effector
	X	Absolutely inhibitory
	?	Unknown (treated as no effect)
Boolean operators:	AND	Used when several states are required for a certain effect (Intersection of states)
	OR	Used when several states can give an effect individually (Union of states)

The rxncon toolbox

The rxncon toolbox is developed open source under the lGPL license and can be downloaded from http://rxncon.org or directly form http://sourceforge.net/projects/yeastmap/. It is browser based, but is run locally. Installation guides are available at http://rxncon.org/rxncon/default/tutorial.

Network reconstruction

We collected two classes of information during the literature curation: Mechanistic information in terms of elemental reactions and contingencies, and physiological/functional information in terms of input-output relationships. The first was used for the actual network reconstruction, and the second for the network validation. Hence, we used qualitatively different data to build and to validate the network reconstruction.

The mechanistic information was divided into reactions and contingencies. We can exemplify this with the relationship between Snf1 and Mig1: Snf1 phosphorylation leads to nuclear export of Mig1. This is actually three different statements, each of which requires its own evidence. First, Snf1 phosphorylates Mig1. Second, the nuclear pore complex export Mig1. Third, the nuclear export only happens when Mig1 is phosphorylated. The first two statements are reactions, and can be written in rxncon short notation (as they appear in figure 2) as:

1. Snf1_P+_Mig1
2. NPC_NEXP_Mig1

The product states of these reactions are Mig1-{P} and Mig1-{Cytoplasm}. Not that these states define to possibly overlapping set; the intersection of these two states would define the cytoplasmic, phosphorylated form of Mig1. Only reaction 2 has a source state, Mig1-{Nuclear}, which is consumed by the second reaction. With these pieces, we can now define the contingency:

3. NPC_NEXP_Mig1 ! Mig1-{P}

Meaning that reaction 3 (NPC_NEXP_Mig1) requires (!) Mig1 to be phosphorylated (Mig1-{P}).

The physiological/functional information consists of inputs/output relationships that are known to require Snf1. For example, we know that glucose depletion relieves repression of Mig1 regulated

genes via Snf1. Hence, a functional model should, upon changes in glucose, convey these changes to the Mig1 regulated genes.

We took a conservative approach to the network reconstruction. The network reconstruction is based on direct mechanistic connections between components, which must be possible to define in terms of reactions or contingencies as defined above. That means that genetic data alone is of limited use, due to the possibility of indirect effects. It also means that we re-evaluated the evidence presented in the papers, rather than relying on the often highly accurate but sometimes speculative interpretation of the authors. Hence, we could not make use of review papers. This conservative angle led us to exclude some components that are likely to be part of the extended Snf1 network based on the lack of direct mechanistic evidence.

The most prominent example is Adr1: Adr1 is phosphorylated on Ser230 in a Snf1 dependent manner (4). However, the mechanism of this interaction is unknown. The interaction between Adr1 and Snf1 could be direct since they have been found to co-localise (5). However, since the *snf1*Δ mutant shows the opposite transcription phenotype to the Adr1^{S230A} mutant, it is unlikely that Snf1 is the kinase of Adr1 (4). Moreover, deletion of Snf1 increases the phosphorylation level of Adr1 on Ser230, indicating that Snf1 rather activates a phosphatase or inactivates a protein kinase under glucose limited conditions than directly phosphorylates Adr1 (6). Undoubtedly, literature evidence shows that Snf1 regulates the transcription factor Adr1, however the exact mechanism and its components are unknown and therefore we did not include it in the network reconstruction.

The mechanistic information was entered into the reaction and contingency list tabs of the Excel based rxncon network definition (Sup. file 1 and 2), together with annotations and references.

Network visualisation

The network graphs were generated automatically using the *rxncon* software and visualised in Cytoscape (1, 7). The reaction graph (Fig 1) visualises the network components and the reactions between them at a topological level. Hence, it represents mechanistic connection and defines all possible information paths, but omits any information of causality. This representation is hence incomplete, but useful to get an overview of the pathway components and their connections.

In contrast, the regulatory graph (e.g. Fig 2) visualises the information flow between elemental reactions and states, i.e., how reactions produce or consume states, and how states stimulate or inhibit reactions via contingencies. The reaction-to-state edges define which reactions produce or consume which states, corresponding to the information in the reaction graph (Fig 1). The state-to-reaction edges define how states influence reactions, and hence define the causal relationship missing in the reaction graph. For a network to transmit information, it must be connected in the regulatory graph. However, this is not sufficient, as it must be possible both to turn on and shut off reactions and states that are dynamically responsive to the signal.

Model generation and simulation

The rxncon network uniquely defines a bipartite Boolean model (bBM) with one specific truth table. The bBM is based on the regulatory graph and, hence, captures the information flow through the

pathway. We generated the bBM with the rxncon software, simulated it using the built-in Boolean simulator and visualised the attractor states on the regulatory graph in Cytoscape (8). The model generation is automatic, based on the network reconstruction. However, to simulate it, we adapted the initial conditions. By default, all components are TRUE (wild-type; no regulated expression of pathway components), and all reactions and states start as FALSE. However, we needed to seed localisation of all components that are translocated; Snf1 (cytosol), Gln3 (cytosol), Msn2 (cytosol), Mig1 (nucleus) and Hxk2 (both nucleus and cytosol), to mimic cells grown in glucose without stress (our default condition). Before model generation, we also eliminated the mutual exclusivity in the bindings between Gal83--Snf1, Sip1--Snf1, and Sip2--Snf1, as well as Glc7--Reg1 and Glc7--Reg2. These bindings are mutually exclusive on the level of individual proteins, but lead to artificial cyclic attractors in the Boolean model that have no informational value.

We ran the simulations with these settings and synchronous updating in the Boolean network modelling software BooleanNet (9). It provides a set of functions for the simulation of biological regulatory networks in a Boolean formalism and is integrated in the rxncon bBM simulator. The Boolean simulations that went beyond the elementary input/output analyses (e.g. automated searches for multiple attractor states) were performed with custom made scripts implemented in the Python programming language calling the BooleanNet software library directly.

Network validation and gap filling

The gap filling process was done by manual evaluation and adaptation of the bBM. We simulated the bBM with a given input configuration until the attractor state was reached, and compared this to the expected simulation outcome (Table 1). All listed output should match the expected state (ON/OFF) when the input conditions are as specified, and should have the opposite state on glucose without stress. When the output did not show the expected behaviour, we manually examined the network to see where the signal was stuck: This would be between the nodes that changed according to the input, and the nodes that did not but were expected to. When necessary, we adapted the network definition to resolve blocks and/or constitutive activities as detailed in the results section. All such adaptations have been clearly labelled as hypotheses in the updated network definition (Sup. file 2).

Analysis of initial conditions for the bBM

We also analysed the impact of the initial settings. We could not perform an exhaustive search, as the number of reactions and states are 72 and 64, respectively, corresponding to 2^{136} (more than 10^{40}) possible starting conditions. However, we reversed the initial settings (all states TRUE, all reactions TRUE), and scanned both settings for changes in individual states (i.e. all but one state/reaction TRUE or FALSE). We tested both these sets in the presence and absence of glucose, for a total of 544 simulations. All these simulations converged on two attractors, each appearing 272 times, depending on whether glucose was present or not (stress perturbations were not considered in this search). Finally, we mimicked the *snf1*Δ mutant by setting the Snf1 node to FALSE. In this last test, none of the input-output relationships of the NR2 were functional, as expected as all signals pass through the Snf1 complex.

Generation of the rule-based model

We used the rxncon toolbox to generate a rule-based model in the BioNetGen language (BNGL) (10). The generation of the rule-based model is in principle automated. However, the reaction types are hard-coded in the current rxncon-to-BNGL converter, and we have a number of reaction types in the Snf1 network that was not foreseen in the original implementation. The implementation of a generic translation engine is in progress, but for this study we used a work-around to generate the BNGL model: We changed acetylation, (de)sumoylation and (de)ubiquitylation reactions to (de)phosphorylation reactions in the network definition and instead named the residues accordingly (Acetylation, SUMOylation, Ubiquitylation), which enabled us to use the automatic export function in rxncon (Get Source Code, under the Model tab). Functionally, this makes no difference. The export generated a model with trivial parameters (all 1) and initial amounts (all 100), which has been formatted for and tested in NFsim (11). The model has 176 parameters, making parameter estimation a massively underdetermined problem.

Supplementary references

1. Tiger CF, Krause F, Cedersund G, Palmer R, Klipp E, Hohmann S, et al. A framework for mapping, visualisation and automatic model creation of signal-transduction networks. Mol Syst Biol. 2012;8:578. Epub 2012/04/26.
2. Le Novere N, Hucka M, Mi H, Moodie S, Schreiber F, Sorokin A, et al. The Systems Biology Graphical Notation. Nature biotechnology. 2009;27(8):735-41. Epub 2009/08/12.
3. Chatr-Aryamontri A, Breitkreutz BJ, Oughtred R, Boucher L, Heinicke S, Chen D, et al. The BioGRID interaction database: 2015 update. Nucleic acids research. 2015;43(Database issue):D470-8. Epub 2014/11/28.
4. Ratnakumar S, Young ET. Snf1 dependence of peroxisomal gene expression is mediated by Adr1. The Journal of biological chemistry. 2010;285(14):10703-14. Epub 2010/02/09.
5. Young ET, Kacherovsky N, Van Riper K. Snf1 protein kinase regulates Adr1 binding to chromatin but not transcription activation. The Journal of biological chemistry. 2002;277(41):38095-103. Epub 2002/08/09.
6. Ratnakumar S, Kacherovsky N, Arms E, Young ET. Snf1 controls the activity of adr1 through dephosphorylation of Ser230. Genetics. 2009;182(3):735-45. Epub 2009/04/29.
7. Shannon P, Markiel A, Ozier O, Baliga NS, Wang JT, Ramage D, et al. Cytoscape: a software environment for integrated models of biomolecular interaction networks. Genome Res. 2003;13(11):2498-504. Epub 2003/11/05.
8. Flottmann M, Krause F, Klipp E, Krantz M. Reaction-contingency based bipartite Boolean modelling. BMC Syst Biol. 2013;7:58. Epub 2013/07/10.
9. Albert I, Thakar J, Li S, Zhang R, Albert R. Boolean network simulations for life scientists. Source code for biology and medicine. 2008;3:16. Epub 2008/11/19.
10. Blinov ML, Faeder JR, Goldstein B, Hlavacek WS. BioNetGen: software for rule-based modeling of signal transduction based on the interactions of molecular domains. Bioinformatics (Oxford, England). 2004;20(17):3289-91.
11. Sneddon MW, Faeder JR, Emonet T. Efficient modeling, simulation and coarse-graining of biological complexity with NFsim. Nat Methods. 2011;8(2):177-83. Epub 2010/12/28.
