# Supplementary material for: Network reconstruction and validation of the Snf1/AMPK pathway in baker’s yeast based on a comprehensive literature review
Source: NPJ Syst Biol Appl. 2015 Oct 22;1:15007–. doi: 10.1038/npjsba.2015.7 (PMC5516868; doi:10.1038/npjsba.2015.7)
Supplement: Supplementary Information [file npjsba20157-s2.doc]

**Supplementary file 1: The initial network reconstruction.** The NR1 network file in the rxncon format, which is described in detail elsewhere (11).

**Supplementary file 2: The final network reconstruction.** The NR2 network file in the rxncon format, which is described in detail elsewhere (11).

**Supplementary file 3: The Snf1 literature.** This table lists all papers evaluated as part of this reconstruction.

**Supplementary file 4: NR2 as BNGL model for simulation in BioNetGen or NFsim**

**Supplementary file 5: NR1 as cytoscape file.** This file can be opened with Cytoscape 3 (http://cytoscape.org/), but not with older versions.

**Supplementary file 6: NR2 as cytoscape file.** This file can be opened with Cytoscape 3 (http://cytoscape.org/), but not with older versions.

**Supplementary file 7: NR2 as BooleanNet file for simulation in Python**

**Supplementary file 8: NR2 as BoolNet file for simulation in R**

**Supplementary file 9: The steady state of the Snf1 bBM in the presence of glucose is a point attractor.** The end state of the bBM simulation in the presence of glucose visualised on the regulatory graph of the updated network. The pale nodes are inactive and the filled nodes are active.

**Supplementary file 10: The steady state of the Snf1 bBM under salt stress is a point attractor.** The end state of the bBM simulation under salt stress visualised on the regulatory graph of the updated network. The pale nodes are inactive and the filled nodes are active.

**Supplementary file 11: The steady state of the Snf1 bBM in alkaline conditions is a point attractor.** The end state of the bBM simulation in alkaline conditions visualised on the regulatory graph of the updated network. The pale nodes are inactive and the filled nodes are active.

**Supplementary file 12: The steady state of the Snf1 bBM under nitrogen starvation is a point attractor.** The end state of the bBM simulation under nitrogen starvation visualised on the regulatory graph of the updated network. The pale nodes are inactive and the filled nodes are active.
