# Supplementary figures and images for: Network reconstruction and validation of the Snf1/AMPK pathway in baker’s yeast based on a comprehensive literature review
Source: NPJ Syst Biol Appl. 2015 Oct 22;1:15007–. doi: 10.1038/npjsba.2015.7 (PMC5516868; doi:10.1038/npjsba.2015.7)

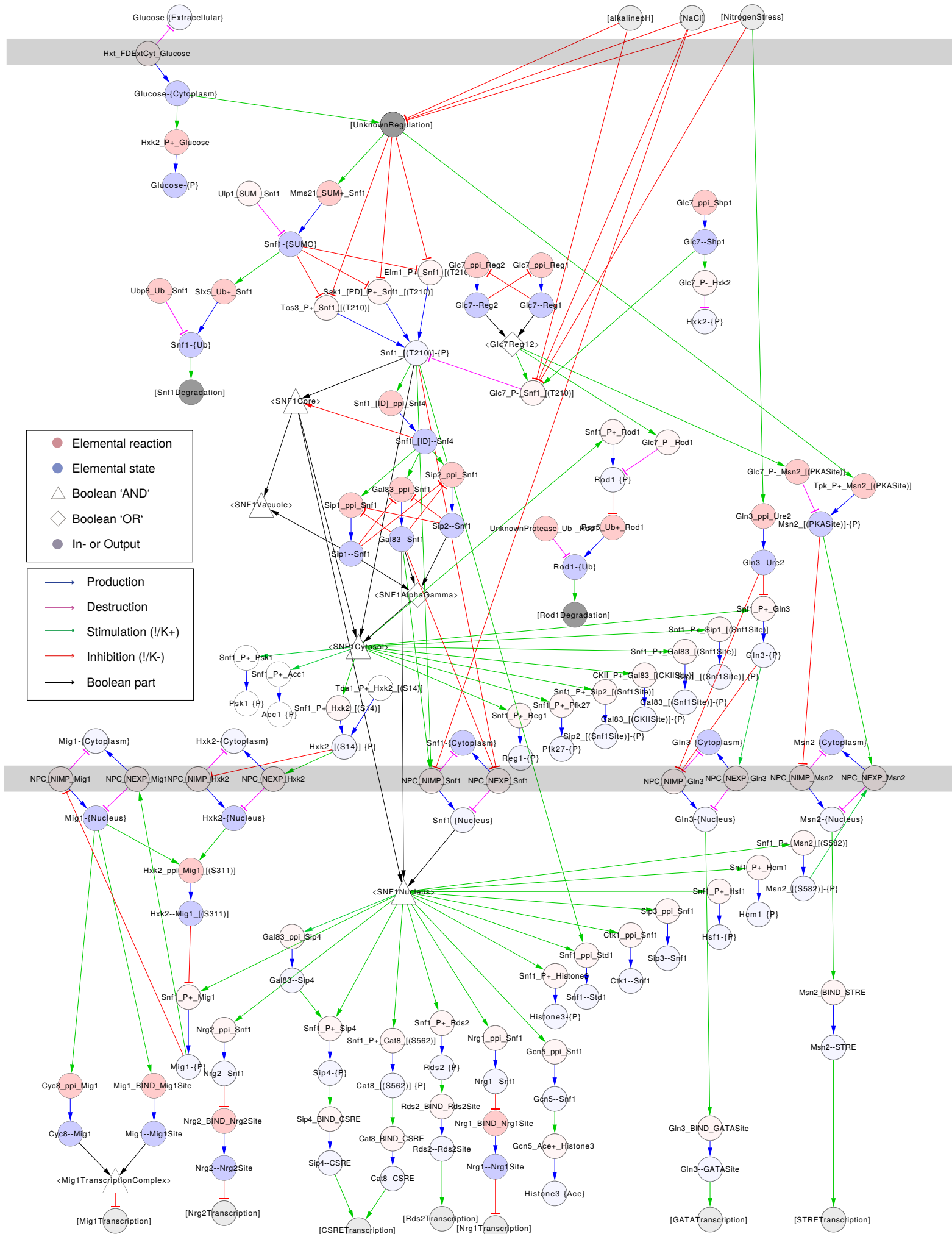

Supplement: Supplementary Figure S1 [file npjsba20157-s3.pdf]

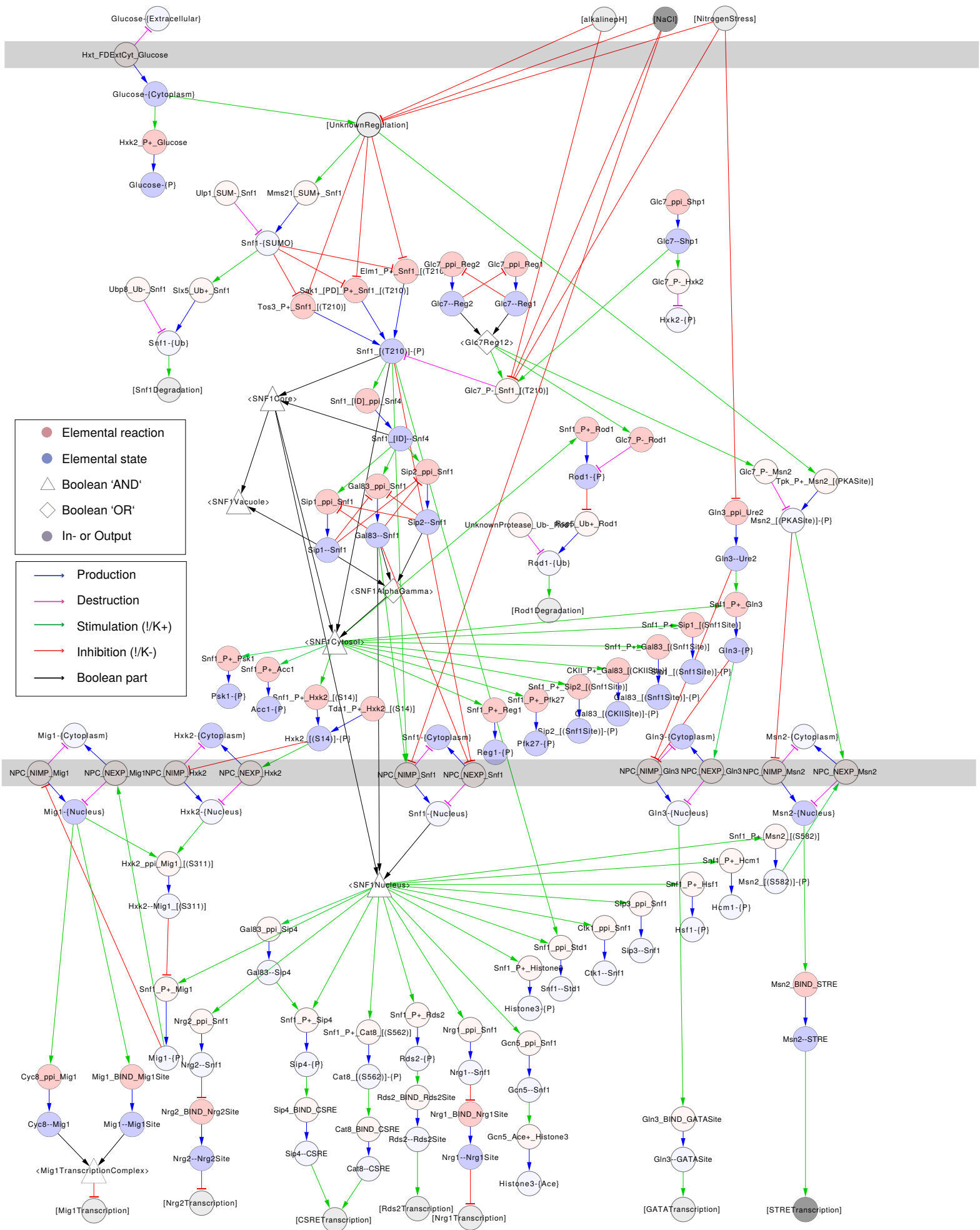

Supplement: Supplementary Figure S2 [file npjsba20157-s4.pdf]

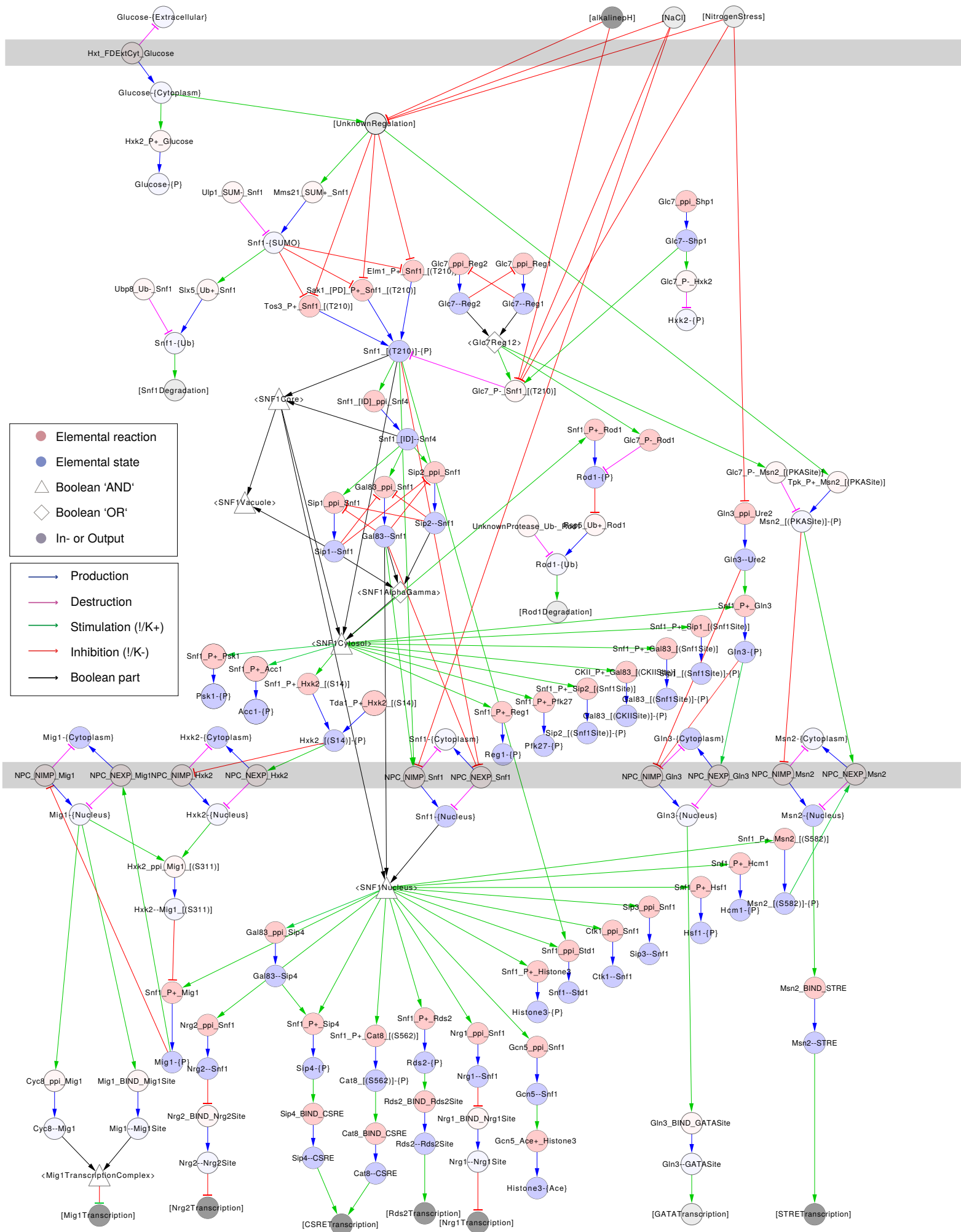

Supplement: Supplementary Figure S3 [file npjsba20157-s5.pdf]

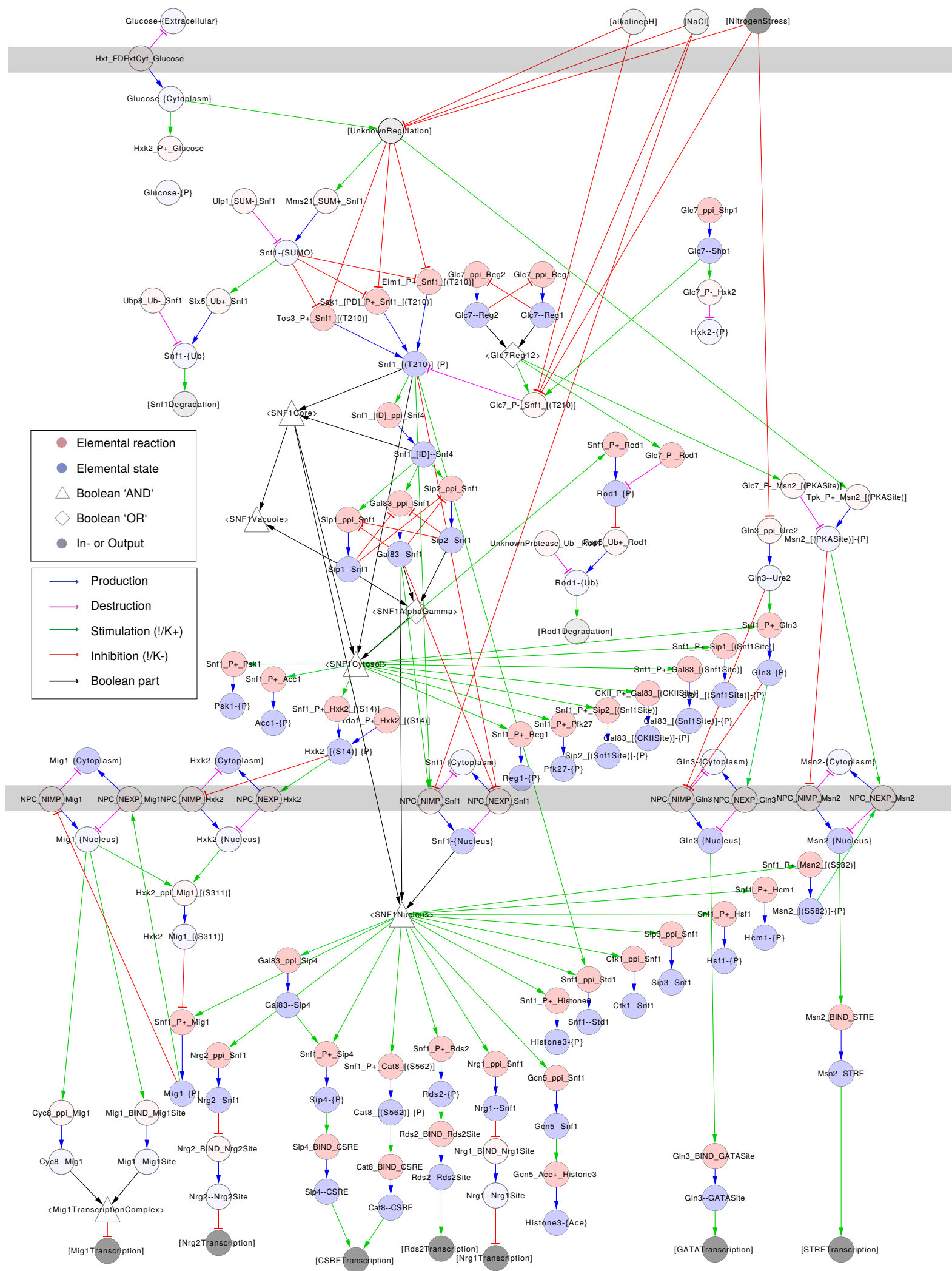

Supplement: Supplementary Figure S4 [file npjsba20157-s6.pdf]
