# Supplementary material for: Network reconstruction and validation of the Snf1/AMPK pathway in baker’s yeast based on a comprehensive literature review
Source: NPJ Syst Biol Appl. 2015 Oct 22;1:15007–. doi: 10.1038/npjsba.2015.7 (PMC5516868; doi:10.1038/npjsba.2015.7)
Supplement: Supplementary Table S3 [file npjsba20157-s9.pdf]

Title	Author	Year	Pubmed ID
Genetics of carbon catabolite repression in Saccharomyces cerevisiae: genes involved in the derepression process	Zimmermann et al.	1977	194140
Isolation and characterization of yeast mutants defective in intermediary carbon metabolism and in carbon catabolite derepression	Ciriacy et al.	1977	197391
Mutants of yeast defective in sucrose utilization	Carlson et al.	1981	7040163
Two differentially regulated mRNAs with different 5' ends encode secreted with intracellular forms of yeast invertase	Carlson et al.	1982	7039847
Structure and expression of the SNF1 gene of Saccharomyces cerevisiae	Celenza et al.	1984	6366513
Cloning and genetic mapping of SNF1, a gene required for expression of glucose-repressible genes in Saccharomyces cerevisiae	Celenza et al.	1984	6366512
Identification of new genes involved in the regulation of yeast alcohol dehydrogenase II	Denis et al.	1984	6392016
A suppressor of SNF1 mutations causes constitutive high-level invertase synthesis in yeast	Carlson et al.	1984	6373495
Genes affecting the regulation of SUC2 gene expression by glucose repression in Saccharomyces cerevisiae	Neigeborn et al.	1984	6392017
Upstream region of the SUC2 gene confers regulated expression to a heterologous gene in Saccharomyces cerevisiae	Sarokin et al.	1985	3939253
Upstream region of the SUC2 gene confers regulated expression to a heterologous gene in Saccharomyces cerevisiae	Sarokin et al.	1985	3939253
A yeast gene that is essential for release from glucose repression encodes a protein kinase	Celenza et al.	1986	3526554
Molecular analysis of SSN6, a gene functionally related to the SNF1 protein kinase of Saccharomyces cerevisiae	Schultz et al.	1987	3316983
High-affinity glucose transport in Saccharomyces cerevisiae is under general glucose repression control	Bisson et al.	1988	3049551
Isolation and characterization of mutants which show an oversecretion phenotype in Saccharomyces cerevisiae	Sakai et al.	1988	3042508
Control of the Saccharomyces cerevisiae regulatory gene PET494: transcriptional repression by glucose and translational induction by oxygen	Marykwas et al.	1989	2540420
Mutational analysis of the Saccharomyces cerevisiae SNF1 protein kinase and evidence for functional interaction with the SNF4 protein	Celenza et al.	1989	2557546
Molecular Analysis of the SNF4 Gene of Saccharomyces cerevisiae : Evidence for Physical Association of the SNF4 Protein with the SNF1 Protein Kinase	Celenza et al.	1989	2481228
A novel genetic system to detect protein-protein interactions	Fields et al.	1989	2547163
Yeast MIG1 repressor is related to the mammalian early growth response and Wilms' tumour finger proteins.	Nehlin et al.	1990	2167835
Release of two Saccharomyces cerevisiae cytochrome genes, COX6 and CYC1, from glucose repression requires the SNF1 and SSN6 gene products	Wright et al.	1990	2154683
The N-terminal TPR region is the functional domain of SSN6, a nuclear phosphoprotein of Saccharomyces cerevisiae	Schultz et al.	1990	2201901
Consequences of growth media, gene copy number, and regulatory mutations on the expression of the PRB1 gene of Saccharomyces cerevisiae	Moehle et al.	1990	2407604
Two systems of glucose repression of the GAL1 promoter in Saccharomyces cerevisiae	Flick et al.	1990	2201902
Increased dosage of the MSN1 gene restores invertase expression in yeast mutants defective in the SNF1 protein kinase	Estruch et al.	1990	2263457
Characterization of TUP1, a mediator of glucose repression in Saccharomyces cerevisiae	Williams et al.	1990	2247069
Complementation of snf1, a mutation affecting global regulation of carbon metabolism in yeast, by a plant protein kinase cDNA	Alderson et al.	1991	1924320
The CCR1 (SNF1) and SCH9 protein kinases act independently of cAMP-dependent protein kinase and the transcriptional activator ADR1 in controlling yeast ADH2 expression	Denis et al.	1991	1944227
Extragenic Suppressors of Yeast Glucose Derepression Mutants Leading to Constitutive Synthesis of Several Glucose-Repressible Enzymes	Schüller et al.	1991	2002006
Deletion of SNF1 affects the nutrient response of yeast and resembles mutations which activate the adenylate cyclase pathway	Thompson-Jaeger et al.	1991	1752415
A protein kinase substrate identified by the Two-hybrid system	Yang et al.	1992	1496382
Yeast casein kinase I homologues: an essential gene pair	Robinson et al.	1992	1729698
N-terminal mutations modulate yeast SNF1 protein kinase function	Estruch et al.	1992	1468623
Analysis of URSG-mediated glucose repression of the GAL1 promoter of Saccharomyces cerevisiae	Flick et al.	1992	1541392
Two complementary approaches to study peroxisome biogenesis in Saccharomyces cerevisiae: forward and reversed genetics	Kunau et al.	1992	1444337
ABF1 is a phosphoprotein and plays a role in carbon source control of COX6 transcription in Saccharomyces cerevisiae	Silve et al.	1992	1324416
Relationship of the cAMP-dependent protein kinase pathway to the SNF1 protein kinase and invertase expression in Saccharomyces cerevisiae	Hubbard et al.	1992	1310088
Multiple mechanisms mediate glucose repression of the yeast GAL1 gene	Lamphier et al.	1992	1631075
Chromatin structure of the yeast SUC2 promoter in regulatory mutants	Matallana et al.	1992	1538695
Regulation of yeast COX6 by the general transcription factor ABF1 and separate HAP2- and heme-responsive elements	Trawick et al.	1992	1314953
Control of peroxisome proliferation in Saccharomyces cerevisiae by ADR1, SNF1 (CAT1, CCR1) and SNF4 (CAT3).	Simon et al.	1992	1355328
Isolation of Peroxisome Assembly Mutants from Saccharomyces cerevisiae with Different Morphologies Using a Novel Selection Procedure	Leij et al.	1992	1356111
Genetic and molecular characterization of GAL83: its interaction and similarities with other genes involved in glucose repression in Sacc	Erickson et al.	1993	8293971
Complex formation between RAS and RAF and other protein kinases	Van Aelst et al.	1993	8327501
Transcriptional regulation of the isocitrate lyase encoding gene in Saccharomyces cerevisiae	Fernandez et al.	1993	8224185
The YAL017 gene on the left arm of chromosome I of Saccharomyces cerevisiae encodes a putative serine/threonine protein kinase	Clark et al.	1993	8322517
DNA sequence analysis of a 17 kb fragment of yeast chromosome XI physically localizes the MRB1 gene and reveals eight new open reading frames, including a homologue of the KIN1/KIN2 and SNF1 protein kinases	Pallier et al.	1993	8256524
Genes required for derepression of an extracellular glucoamylase gene, STA2, in the yeast Saccharomyces	Kuchin et al.	1993	8322516
Molecular and genetic analysis of the SNF7 gene in Saccharomyces cerevisiae	Tu et al.	1993	8224817
Two homologous zinc finger genes identified by multicopy suppression in a SNF1 protein kinase mutant of Saccharomyces cerevisiae	Estruch et al.	1993	8321194
A Saccharomyces cerevisiae upstream activating sequence mediates induction of peroxisome proliferation by fatty acids	Filipits et al.	1993	8406042
Yeast SNF1 is functionally related to mammalian AMP-activated protein kinase and regulates acetyl-CoA carboxylase in vivo	Woods et al.	1994	7913470
The GLC7 type 1 protein phosphatase is required for glucose repression in Saccharomyces cerevisiae	Tu et al.	1994	7935396
A family of proteins containing a conserved domain that mediates interaction with the yeast SNF1 protein kinase complex	Yang et al.	1994	7813428
Synergistic release from glucose repression by mig1 and ssn mutations in Saccharomyces cerevisiae	Vallier et al.	1994	8056322
Mammalian AMP-activated protein kinase is homologous to yeast and plant protein kinases involved in the regulation of carbon metabolism	Carling et al.	1994	7908907
Characterization and chromosomal localization of the human homologue of a rat AMP-activated protein kinase-encoding gene: a major regulator of lipid metabolism in mammals	Aguan et al.	1994	7959015
Characterization of the osmotic-stress response in Saccharomyces cerevisiae: osmotic stress and glucose repression regulate glycerol-3-phosphate dehydrogenase independently	Albertyn et al.	1994	8082159
Heat shock transcription factor activates yeast metallothionein gene expression in response to heat and glucose starvation via distinct signalling pathways	Tamai et al.	1994	7969152
Adr1 and Snf1 mediate different mechanisms in transcriptional regulation of yeast POT1 gene	Navarro et al.	1994	7914081
Characterization of glycogen-deficient glc mutants of Saccharomyces Cerevisiae	Cannon et al.	1994	8150278
Identification and characterization of a novel yeast gene: the YGP1 gene product is a highly glycosylated secreted protein that is synthesized in response to nutrient limitation	Destruelle et al.	1994	8139573
Characterization of tobacco protein kinase NPK5, a homolog of Saccharomyces Cerevisae SNF1 that constitutively activates expression of the glucose-repressible SUC2 gene for a secreted invertase of S. cerevisiae	Muranaka et al.	1994	8164654
Glucose repression of yeast mitochondrial transcription: kinetics of derepression and role of nuclear genes	Ulery et al.	1994	8289797
Molecular physiology. Ways of coping with stress	Hardie et al.	1994	8065446
Interactions between cAMP-dependent and SNF1 protein kinases in the control of glycogen accumulation in Saccharomyces cerevisiae	Hardy et al.	1994	7961723
Dosage-dependent modulation of glucose repression by MSN3 (STD1) in Saccharomyces cerevisiae	Hubbard et al.	1994	8114728
A carbon source-responsive promoter element necessary for activation of the isocitrate lyase gene ICL1 is common to genes of the gluconeogenic pathway in the yeast Saccharomyces cerevisiae	Schöler et al.	1994	8196607
Analysis of the SIP3 protein identified in a two-hybrid screen for interaction with the SNF1 protein kinase	Lesage et al.	1994	8127709
Characterization of a unique protein component of yeast RNase MRP: an RNA-binding protein with a zinc-cluster domain	Schmitt et al.	1994	7958920
Multiple mechanisms provide rapid and stringent glucose repression of GAL gene expression in Saccharomyces cerevisiae	Johnston et al.	1994	8196626
Mammalian AMP-activated protein kinase shares structural and functional homology with the catalytic domain of yeast Snf1 protein kinase	Mitchelhill et al.	1994	7905477
SIP1 is a catabolite repression-specific negative regulator of GAL gene expression	Mylin et al.	1994	8088514
Expression of high-affinity glucose transport protein Hxt2p of Saccharomyces cerevisiae is both repressed and induced by glucose and appears to be regulated posttranslationally	Wendell et al.	1994	8206851
Genetic and carbon source regulation of phosphorylation of Sip1p, a Snf1p-associated protein involved in carbon response in Saccharomyces cerevisiae	Long et al.	1995	7785324
Repression by SSN6-TUP1 is directed by MIG1, a repressor/activator protein	Treitel et al.	1995	7724528
CAT8, a new zinc cluster-encoding gene necessary for derepression of gluconeogenic enzymes in the yeast Saccharomyces cerevisiae	Hedges et al.	1995	7891685
REG1 binds to protein phosphatase type 1 and regulates glucose repression in Saccharomyces cerevisiae	Tu, Carlson	1995	8846786
STD1 (MSN3) interacts directly with the TATA-binding protein and modulates transcription of the SUC2 gene of Saccharomyces cerevisiae	Tillman et al.	1995	7667094
Similar substrate recognition motifs for mammalian AMP-activated protein kinase, higher plant HMG-CoA reductase kinase-A, yeast SNF1, and mammalian calmodulin-dependent protein kinase I	Dale et al.	1995	7698321
Sequence analysis of a 44 kb DNA fragment of yeast chromosome XV including the Tyl-H3 retrotransposon, the suf1(+) frameshift suppressor gene for tRNA-Gly, the yeast transfer RNA-Thr-1a and a delta element	Vandenbol et al.	1995	7502582
Cyclin-dependent protein kinase and cyclin homologs SSN3 and SSN8 contribute to transcriptional control in yeast	Kuchin et al.	1995	7732022
CAT5, a new gene necessary for derepression of gluconeogenic enzymes in Saccharomyces cerevisiae	Proft et al.	1995	8557031
Molecular analysis of the SNF8 gene of Saccharomyces cerevisiae	Yeghiayan et al.	1995	7785322
Expression of the FOX1 gene of Saccharomyces cerevisiae is regulated by carbon source, but not by the known glucose repression genes	Stanway et al.	1995	7586025
The glucose repression and RAS-cAMP signal transduction pathways of Saccharomyces cerevisiae each affect RNA processing and the synthesis of a reporter protein	Tung et al.	1995	7715603
The Saccharomyces cerevisiae zinc finger proteins Msn2p and Msn4p are required for transcriptional induction through the stress-response element (STRE)	Martinez-Pastor et al.	1996	8641288
The REG2 gene of Saccharomyces cerevisiae encodes a type 1 protein phosphatase-binding protein that functions with Reg1p and the Snf1 protein kinase to regulate growth	Frederick et al.	1996	8649403
Characterization of AMP-activated protein kinase beta and gamma subunits. Assembly of the heterotrimeric complex in vitro	Woods et al.	1996	8626596
Identification and characterisation of two transcriptional repressor elements within the coding sequence of the Saccharomyces cerevisiae HXK2 gene	Herrero et al	1996	8657561
Functional domains in the Mig1 repressor	Ostling et al.	1996	8622676
Yeast SNF1 Protein Kinase Interacts with SIP4 , a C 6 Zinc Cluster Transcriptional Activator : a New Role for SNF1 in the Glucose Response	Lesage et al.	1996	8628258
Genetic interactions between REG1/HEX2 and GLC7, the gene encoding the protein phosphatase type 1 catalytic subunit in Saccharomy	Huang et al	1996	8722767
FOG1 and FOG2 genes, required for the transcriptional activation of glucose-repressible genes of Kluyveromyces lactis , are homologous to GAL83 and SNF1 of Saccharomyces cerevisiae	Goffrini et al.	1996	8598052
Glucose regulates protein interactions within the yeast SNF1 protein kinase complex.	Jiang et al.	1996	8985180
Dual influence of the yeast Cat1p (Snf1p) protein kinase on carbon source-dependent transcriptional activation of gluconeogenic genes by the regulatory gene CAT8	Rahner et al.	1996	8710504
Pho85p, a cyclin-dependent protein kinase, and the Snf1p protein kinase act antagonistically to control glycogen accumulation in Saccharomyces cerevisiae	Huang et al.	1996	8754836
Cloning and characterization of a novel serine/threonine protein kinase expressed in early Xenopus embryos	Su et al.	1996	8662877
Non-catalytic beta- and gamma-subunit isoforms of the 5'-AMP-activated protein kinase	Gao et al.	1996	8621499
Glucose repression/derepression in budding yeast: SNF1 protein kinase is activated by phosphorylation under derepressing conditions, and this correlates with a high AMP:ATP ratio.	Wilson et al.	1996	8939604
Disruption of the SNF1 gene abolishes trehalose utilization in the pathogenic yeast Candida glabrata	Petter et al.	1996	8945576
Glucose repression affects ion homeostasis in yeast through the regulation of the stress-activated ENA1 gene	Alepuz et al.	1997	9383192
The Snf1 protein kinase and its activating subunit, Snf4, interact with distinct domains of the Sip1/Sip2/Gal83 component in the kinase complex	Jiang et al.	1997	9121458
Glucose represses the lactose-galactose regulon in Kluyveromyces lactis through a SNF1 and MIG1- dependent pathway that modulates galactokinase (GAL1) gene expression	Dong et al.	1997	9278487
Comparative analysis in three fungi reveals structurally and functionally conserved regions in the Mig1 repressor	Cassart et al.	1997	9230894
expression of the suc2 gene of Saccharomyces Cerevisiae is induced by low levels of glucose	Özcan et al.	1997	9046094
Derepression of gene expression mediated by the 5' upstream region of the isocitrate lyase gene of Candida tropicalis is controlled by two distinct regulatory pathways in Saccharomyces cerevisiae	Umemura et al.	1997	9057841
Analysis of carbon source-regulated gene expression by the upstream region of the Candida tropicalis malate synthase gene in Saccharomyces cerevisiae	Umemura et al.	1997	9003461
Glucose-6-P control of glycogen synthase phosphorylation in yeast	Huang et al.	1997	9278401
Regulation of the proteinase B structural gene PRB1 in Saccharomyces cerevisiae	Naik et al.	1997	9045801
A gene homologous to Saccharomyces cerevisiae SNF1 appears to be essential for the viability of Candida albicans	Petter et al.	1997	9393775
Mutations in GSF1 and GSF2 alter glucose signaling in Saccharomyces cerevisiae	Sherwood et al.	1997	9335593
Glucose Derepression of Gluconeogenic Enzymes in Saccharomyces cerevisiae Correlates with Phosphorylation of the Gene Activator Cat8p	Randez-Gil et al.	1997	9111319
Efficient signal transduction by a chimeric yeast-mammalian G protein alpha subunit Gpa1-Gsalpha covalently fused to the yeast receptor Ste2	Medici et al.	1997	9405353
Glucose-regulated interaction of a regulatory subunit of protein phosphatase 1 with the Snf1 protein kinase in Saccharomyces cerevisiae	Ludin et al.	1998	9600950
Multiple regulatory proteins mediate repression and activation by interaction with the yeast Mig1 binding site	Wu et al.	1998	9730278
Snf1 protein kinase regulates phosphorylation of the Mig1 repressor in Saccharomyces cerevisiae	Treitel et al.	1998	9774644

Characterization of three related glucose repressors and genes they regulate in Saccharomyces cerevisiae	Lutfiyya et al.	1998	9832517
Negative control of the Mig1p repressor by Snf1p-dependent phosphorylation in the absence of glucose	Ostling et al.	1998	9523726
Insulin Signaling in the Yeast Saccharomyces cereisiae. 1, stimulation of glucose metabolism and Snf1 kinase by human insulin	Muller et al.	1998	9628731
Carbon source-dependent phosphorylation of hexokinase PII and its role in the glucose-signaling response in yeast	Randez-Gil et al.	1998	9566913
Negative control of the Mig1p repressor by Snf1p-dependent phosphorylation in the absence of glucose	Östling et al.	1998	9523726
Catabolite repression mutants of Saccharomyces cerevisiae show altered fermentative metabolism as well as cell cycle behavior in glucose-limited chemostat cultures	Aon et al	1998	10099331
Regulation of glucose utilization in yeast	Carlson	1998	9870704
A 27 kDa protein binds to a positive and a negative regulatory sequence in the promotor of the ICL1 gene from Saccharomyces cerevisiae	Ordiz et al.	1998	9425123
Nuclear localization of the C2H2 zinc finger protein Msn2p is regulated by stress and protein kinase A activity	Görner, Wolfram et al.	1998	9472026
Rice has two distinct classes of protein kinase genes related to SNF1 of Saccharomyces cerevisiae	Takano et al.	1998	9870704
Sip4, a Snf1 kinase-dependent transcriptional activator, binds to the carbon source-responsive element of gluconeogenic genes	Vincent et al.	1998	9843506
A region of the cellobiohydrolase I promoter from the filamentous fungus Trichoderma reesei mediates glucose repression in Saccharomyces cerevisiae, dependent on mitochondrial activity	Carraro et al.	1998	9878550
Amino acid residues in Std1 protein required for induction of SUC2 transcription are also required for suppression of TBPDelta57 growth defect in Saccharomyces cerevisiae	Zhang et al.	1998	9666103
Temperature-induced expression of yeast FKS2 is under the dual control of protein kinase C and calcineurin	Zhao et al.	1998	9447998
Cyclin partners determine Pho85 protein kinase substrate specificity in vitro and in vivo: control of glycogen biosynthesis by Pcl8 and Pcl10	Huang et al	1998	9584169
Snf1 kinase connects nutritional pathways controlling meiosis in Saccharomyces cerevisiae	Honigberg et al.	1998	9671464
Characterization of a Sorghum bicolor gene family encoding putative protein kinases with a high similarity to the yeast SNF1 protein kinase	Annen et al	1998	9484448
Msn2p and Msn4p control a large number of genes induced at the diauxic transition which are repressed by cyclic AMP in Saccharomyces cerevisiae	Boy-Marcotte et al.	1998	9495741
THE AMP-ACTIVATED/SNF1 PROTEIN KINASE SUBFAMILY: Metabolic Sensors of the Eukaryotic Cell?	Hardie et al.	1998	9759505
The nuclear exportin Msn5 is required for nuclear export of the Mig1 glucose repressor of Saccharomyces cerevisiae	De Vit et al.	1999	10556086
Functional Analysis of the Yeast Functional Analysis of the Yeast identifies a protein phosphatase type 1-binding motif as essential for repression of ADH2 expression	Dombek et al.	1999	10454550
Glucose repression in yeast.	Carlson et al.	1999	10322167
Gal83 mediates the interaction of the Snf1 kinase complex with the transcription activator Sip4	Vincent et al.	1999	10581241
The SNF1 kinase complex from Saccharomyces cerevisiae phosphorylates the transcriptional repressor protein Mig1p in vitro at four sites within or near regulatory domain 1	Smith et al.	1999	10403407
The TOR signalling pathway controls nuclear localization of nutrient regulated transcription factors	Beck and Hall	1999	10604478
The Med1 subunit of the yeast mediator complex is involved in both transcriptional activation and repression	Balciunas et al.	1999	9892641
The nucleosome remodeling complex, Snf/Swi, is required for the maintenance of transcription in vivo and is partially redundant with the histone acetyltransferase, Gcn5	Sudarsanam et al.	1999	1171391
Potato StubSNF1 interacts with StubGAL83: a plant protein kinase complex with yeast and mammalian counterparts	Lakatos et al.	1999	10205910
Quantitation of the effects of disruption of catabolite (de)repression genes on the cell cycle behavior of Saccharomyces cerevisiae	Aon et al.	1999	9841784
Expression of the SNF1 gene from candida tropicalis is required for growth on various carbon sources, including glucose	Kana et al.	1999	10525743
Arabidopsis thaliana proteins related to the yeast SIP and SNF4 interact with AKINa1 , an SNF1-like protein kinase	Bouly et al.	1999	10417704
Sequence and phylogenetic analysis of the SNF4/AMPK gamma subunit gene from Drosophila melanogaster	Yoshida et al.	1999	10659773
Regulatory interactions between the Reg1-Glc7 protein phosphatase and the Snf1 protein kinase	Sanz et al.	2000	10648618
Sip5 interacts with both the Reg1/Glc7 protein phosphatase and the Snf1 protein kinase of Saccharomyces cerevisiae	Sanz et al.	2000	10628972
Beta-subunits of Snf1 kinase are required for kinase function and substrate definition	Schmidt et al.	2000	10990457
Analysis of the mechanism by which glucose inhibits maltose induction of MAL gene expression in Saccharomyces	Hu et al.	2000	10628974
Isolation of the MIG1 gene from Candida albicans and effects of its disruption on catabolite repression	Zaragoza et al.	2000	10629176
Regulatory interactions between the Reg1-Glc7 protein phosphatase and the Snf1 protein kinase	Pascual Sanz	2000	10648618
Glucose depletion causes haploid invasive growth in yeast	Cullen et al.	2000	11095711
Functional identification of an Arabidopsis snf4 ortholog by screening for heterologous multicopy suppressors of snf4 deficiency in yeast	Kleinow et al.	2000	10929106
A regulatory shortcut between the Snf1 protein kinase and RNA polymerase II holoenzyme	Kuchin et al.	2000	10869433
Sip2p and its partner snf1p kinase affect aging in S. cerevisiae	Ashrafi et al.	2000	10921902
The Cochliobolus carbonum SNF1 gene is required for cell wall-degrading enzyme expression and virulence on maize	Tonukari et al.	2000	10662860
Cooperative regulation of DOG2, encoding 2-deoxyglucose-6-phosphate phosphatase, by Snf1 kinase and the high-osmolarity glycerol-mitogen-activated protein kinase cascade in stress responses of Saccharomyces cerevisiae	Tsujimoto et al.	2000	10960096
Glucose depletion Rapidly Inhibits Translation Initiation in Yeast	Ashe et al.	2000	10712503
Carbon source-dependent transcriptional regulation of the mitochondrial glycerol-3-phosphate dehydrogenase gene, GUT2, from Saccharomyces cerevisiae	Grauslund et al	2000	11142398
Subcellular localization of the Snf1 kinase is regulated by specific beta subunits and a novel glucose signaling mechanism	Vincent et al.	2001	11331606
Regulation of Snf1 Kinase	McCartney et al.	2001	11486005
Evidence for the involvement of the Glc7-Reg1 phosphatase and the Snf1-Snf4 kinase in the regulation of INO1 transcription in Saccharomyces cerevisiae	Shirra et al	2001	10224244
Snf1--a histone kinase that works in concert with the histone acetyltransferase Gcn5 to regulate transcription.	Lo et al.	2001	11498592
NRG1 is required for glucose repression of the SUC2 and GAL genes of Saccharomyces cerevisiae	Zhou et al.	2001	11281938
Human pancreatic glucokinase (GlkB) complements the glucose signalling defect of Saccharomyces cerevisiae hxk2 mutants	Mayordomo et al	2001	11571755
Regulatory elements in the FBP1 promoter respond differently to glucose-dependent signals in Saccharomyces cerevisiae	Zaragoza et al	2001	11563983
Interaction of the Srb10 kinase with Sip4, a transcriptional activator of gluconeogenic genes in Saccharomyces cerevisiae	Vincent et al.	2001	11486018
Snf1--a histone kinase that works in concert with the histone acetyltransferase Gcn5 to regulate transcription.	Lo et al.	2001	11498592
Contribution of Cat8 and Sip4 to the transcriptional activation of yeast gluconeogenic genes by carbon source-responsive elements	Hiesinger et al	2001	11405098
Three target genes for the transcriptional activator Cat8p of Kluyveromyces lactis: acetyl coenzyme A synthetase genes KlACS1 and KlACS2 and lactate permease gene KlJEN1	Lodi et al	2001	11514507
The yeast cyclins Pc16p and Pc17p are involved in the control of glycogen storage by the cyclin-dependent protein kinase Pho85p	Wang et al	2001	11602261
A transient histone hyperacetylation signal marks nucleosomes for remodeling at the PHO8 promoter in vivo	Reinke et al.	2001	11463378
Interaction of the repressors Nrg1 and Nrg2 with the Snf1 protein kinase in Saccharomyces cerevisiae	Vyas et al.	2001	11404322
SKP1-SnRK protein kinase interactions mediate proteasomal binding of a plant SCF ubiquitin ligase	Farras et al.	2001	11387208
Domain fusion between SNF1-related kinase subunits during plant evolution	Lumbreras et al.	2001	11252725
NRG1 is required for glucose repression of the SUC2 and GAL genes of Saccharomyces cerevisiae	Zhou et al.	2001	11281938
Antagonistic controls of autophagy and glycogen accumulation by Snf1p, the yeast homolog of AMP-activated protein kinase, and the cyclin-dependent kinase Pho85p	Wang et al.	2001	11486014
Inhibition of acetyl coenzyme A carboxylase activity restores expression of the INO1 gene in a snf1 mutant strain of Saccharomyces cerevisiae	Shirra et al.	2001	11486011
Expression and regulation of the AMP-activated protein kinase-SNF1 (sucrose non-fermenting 1) kinase complexes in yeast and mammalian cells: studies using chimaeric catalytic subunits	Daniel et al.	2002	11971761
Snf1 protein kinase and the repressors Nrg1 and Nrg2 regulate FLO11, haploid invasive growth, and diploid pseudohyphal differentiation	Kuchin et al.	2002	12024013
Snf1 protein kinase regulates Adr1 binding to chromatin but not transcription activation.	Young et al.	2002	12167649
Active Snf1 protein kinase inhibits expression of the Saccharomyces cerevisiae HXT1 glucose transporter gene	Tomas-Cobos et al.	2002	12220226
Purification and characterization of Snf1 kinase complexes containing a defined Beta subunit composition.	Nath et al.	2002	12393914
Depression of Saccharomyces cerevisiae invasive growth on non-glucose carbon sources requires the Snf1 kinase	Palecek et al.	2002	12123456
Convergence of the target of rapamycin and the Snf1 protein kinase pathways in the regulation of the subcellular localization of Msn2, a transcriptional activator of STRE (Stress Response Element)-regulated genes.	Mayordomo et al	2002	12093809
Phosphorylation positively regulates DNA binding of the carbon catabolite repressor Cre1 of Hypocrea jecorina (Trichoderma reesei).	Cziferszky et al.	2002	11850429
Constitutively active AMP kinase mutations cause glycogen storage disease mimicking hypertrophic cardiomyopathy	Arad et al	2002	11827995
Transcription activator interactions with multiple SWI/SNF subunits	Neely et al.	2002	11865042
Identification of SnIP1, a novel protein that interacts with SNF1-related protein kinase (SnRK1)	Slocombe et al.	2002	12008897
Convergence of TOR-nitrogen and Snf1-glucose signaling pathways onto Gln3	Bertram et al.	2002	11809814
Meiotic differentiation during colony maturation in Saccharomyces cerevisiae	Purnapatre et al	2002	12420140
The roles of bud-site-selection proteins during haploid invasive growth in yeast	Cullen et al.	2002	12221111
Co-ordinate regulation of lactate metabolism genes in yeast: the role of the lactate permease gene JEN1	Lodi et al.	2002	11810259
The CLN3/SWI6/CLN2 pathway and SNF1 act sequentially to regulate meiotic initiation in Saccharomyces cerevisiae	Purnapatre et al.	2002	12081645
Activation of yeast Snf1 and mammalian AMP-activated protein kinase by upstream kinases	Hong et al.	2003	12847291
Std1p (Msn3p) Positively Regulates the Snf1 Kinase in Saccharomyces cerevisiae	Kuchin et al.	2003	12618390
Yeast Pak1 kinase associates with and activates Snf1	Nath et al.	2003	12748292
Glucose and type 2A protein phosphatase regulate the interaction between catalytic and regulatory subunits of AMP-activated protein kinase	Gimeno-Alcaniz et al.	2003	14516753
Snf1 protein kinase: a key player in the response to cellular stress in yeast	Sanz et al.	2003	12546680
Snf1 kinases with different beta-subunit isoforms play distinct roles in regulating haploid invasive growth	Vyas et al.	2003	12556493
Sip2, an N-myristoylated beta subunit of Snf1 kinase, regulates aging in Saccharomyces cerevisiae by affecting cellular histone kinase activity, recombination at rDNA loci, and silencing	Lin et al.	2003	12562756
Yap1 Accumulates in the Nucleus in Response to Carbon Stress in Saccharomyces cerevisiae	Wiatrowski et al.	2003	12582119
Activation of yeast Snf1 and mammalian AMP-activated protein kinase by upstream kinases	Hong, Carlson	2003	12847291
Elm1p is one of three upstream kinases for the Saccharomyces cerevisiae SNF1 complex.	Sutherland	2003	12906789
Isolation of mutations in the catalytic domain of the snf1 kinase that render its activity independent of the snf4 subunit	Leech et al	2003	12684376
The Snf1 protein kinase controls the induction of genes of the iron uptake pathway at the diauxic shift in Saccharomyces cerevisiae	Haurie et al.	2003	12960168
A computer-based microarray experiment design-system for gene-regulation pathway discovery	Yoo et al.	2003	14728270
New mutations of Saccharomyces cerevisiae that partially relieve both glucose and galactose repression activate the protein kinase Snf1	Rodriguez et al	2003	12702249
Gts1p activates SNF1-dependent derepression of HSP104 and TPS1 in the stationary phase of yeast growth	Yaguchi et al	2003	12782635
Functional diversity of potato SNF1-related kinases tested in Saccharomyces cerevisiae	Lovas	2003	14636999
Abscisic acid and gibberellin differentially regulate expression of genes of the SNF1-related kinase complex in tomato seeds	Bradford et al.	2003	12857836
Characterization of a SNF1 homologue from the phytopathogenic fungus Sclerotinia sclerotiorum	Vacher et al.	2003	12801638
Multiple pathways are co-regulated by the protein kinase Snf1 and the transcription factors Adr1 and Cat8	Young et al.	2003	12676948
Systematic trans-genomic comparison of protein kinases between Arabidopsis and Saccharomyces cerevisiae	Wang et al	2003	12913170
The protein kinase Snf1 is required for tolerance to the ribonucleotide reductase inhibitor hydroxyurea	Dubacq et al.	2004	14993292
Pak1 protein kinase regulates activation and nuclear localization of Snf1-Gal83 protein kinase	Hedbacker et al.	2004	15340085
Sip2, an N-myristoylated beta subunit of Snf1 kinase, regulates aging in Saccharomyces cerevisiae by affecting cellular histone kinase activity, recombination at rDNA loci, and silencing	Hahn et al.	2004	14612437
Cyclic AMP-dependent protein kinase regulates the subcellular localization of Snf1-Sip1 protein kinase	Hedbacker et al.	2004	14966266
Mutations in the gal83 glycogen-binding domain activate the snf1/gal83 kinase pathway by a glycogen-independent mechanism	Wiatrowski et al.	2004	14673168
Cyclic AMP-dependent protein kinase regulates the subcellular localization of Snf1-Sip1 protein kinase	Hedbacker et al.	2004	14966266
The Snf1 kinase controls glucose repression in yeast by modulating interactions between the Mig1 repressor and the Cyc8-Tup1 co-repressor	Papamichos-Chronakis M et al.	2004	15031717
Regulatory network connecting two glucose signal transduction pathways in Saccharomyces cerevisiae	Kaniak et al.	2004	14871952
Transcriptional responses to glucose at different glycolytic rates in Saccharomyces cerevisiae	Elbing et al.	2004	15606773
Transcriptional activators Cat8 and Sip4 discriminate between sequence variants of the carbon source-responsive promoter element in the yeast Saccharomyces cerevisiae.	Roth et al.	2004	14685767
Key Role of Ser562/661 in Snf1-dependent regulation of Cat8p in Saccharomyces cerevisiae and Kluyeromyces lactis	Charbon et al.	2004	15121831
SNF1-related protein kinase (snRK1) phosphorylates class I heat shock protein	Slocombe et al.	2004	15283126
Roles of SWI/SNF and HATs throughout the dynamic transcription of a yeast glucose-repressible gene	Geng et al.	2004	14685262
Carboxylic acids permeases in yeast: two genes in Kluyveromyces lactis	Lodi et al	2004	15363851
Snf1-related protein kinase 1 is needed for growth in a normal day-night light cycle	Thelander et al.	2004	15057278
Biochemical characterization of the tobacco 42-kD protein kinase activated by osmotic stress	Kelner et al.	2004	15466234
Simultaneous yet independent regulation of actin cytoskeletal organization and translation initiation by glucose in Saccharomyces cerevisiae	Uesono et al.	2004	14742701
PLP2/A4 interacts with CCR1 and stimulates migration of CCR1-expressing HOS cells	Lee et al	2004	15474493
Gts1p stabilizes oscillations in energy metabolism by activating the transcription of TPS1 encoding trehalose-6-phosphate synthase 1 in the yeast Saccharomyces	Xu et al	2004	15228382
Dissecting regulatory networks by means of two-dimensional gel electrophoresis: application to the study of the diauxic shift in the yeast Saccharomyces cerevisiae	Haurie et al.	2004	14760706
Yeast Pho85 kinase is required for proper gene expression during the diauxic shift	Nishizawa et al.	2004	15334555
Methionine sulfoximine treatment and carbon starvation elicit Snf1-independent phosphorylation of the transcription activator Gln3 in Saccharomyces cerevisiae	Tate et al.	2004	15911613
A functional analysis reveals dependence on the anaphase-promoting complex for prolonged life span in yeast	Harkness et al	2004	15514051
Combinatorial control by the protein kinases PKA, PHO85 and SNF1 of transcriptional induction of the Saccharomyces cerevisiae GSY2 gene at the diauxic shift	Enjalbert et al.	2004	15221454
Saccharomyces cerevisiae JEN1 promoter activity is inversely related to concentration of repressing sugar	Chambers et al.	2004	14711620
Crystal structure of the protein kinase domain of yeast AMP-activated protein kinase Snf1	Rudolph et al.	2005	16236260
A dual role for PP1 in shaping the Msn2-dependent transcriptional response to glucose starvation	De Wever et al.	2005	16281053

Role of Tos3, a Snf1 protein kinase kinase, during growth of Saccharomyces cerevisiae on nonfermentable carbon sources	Kim et al.	2005	15879520
Snf1 kinase complexes with different beta subunits display stress-dependent preferences for the three Snf1-activating kinases	McCartney et al.	2005	15824893
Function of mammalian LKB1 and Ca2+/calmodulin-dependent protein kinase kinase alpha as Snf1-activating kinases in yeast	Hong et al.	2005	15831494
The Snf1 protein kinase and Sit4 protein phosphatase have opposing functions in regulating TATA-binding protein association with the Saccharomyces cerevisiae INO1 promoter	Shirra et al	2005	15716495
A role for the non-phosphorylated form of yeast Snf1: tolerance to cation and activation of patossium transport	Portillo et al.	2005	15642368
Repressors Nrg1 and Nrg2 regulate a set of stress-responsive genes in Saccharomyces cerevisiae	Vyas et al.	2005	16278455
Histone H3 Ser10 phosphorylation-independent function of Snf1 and Reg1 proteins rescues a gcn5- mutant in HIS3 expression	Liu et al	2005	16287868
Glucose deprivation mediates interaction between CTDK-I and Snf1 in Saccharomyces cerevisiae	Van Driessche et al	2005	16182287
Ca2+/calmodulin-dependent protein kinase kinase-beta acts upstream of AMP-activated protein kinase in mammalian cells	Woods et al.	2005	16054096
SCFGrr1-mediated ubiquitination of Gis4 modulates glucose response in yeast	La rue et al.	2005	15890364
Role of Snf1p in regulation of intracellular sorting of the lactose and galactose transporter Lac12p in Kluyveromyces lactis	Wiedemuth et al.	2005	15821131
Evidence that Spt10 and Spt21 of Saccharomyces cerevisiae play distinct roles in vivo and functionally interact with MCB-binding factor, SCB-binding factor and Snf1	Hess et al	2005	15744051
Carbon Source-dependent assembly of the Snf1p kinase complex in Candida albicans	Corvey et al	2005	15890650
Histone H3 phosphorylation can promote TBP recruitment through distinct promoter-specific mechanisms	Lo et al.	2005	15719021
Regulation of the nucleocytoplasmic distribution of Snf1-Gal83 protein kinase	Hedbacker et al.	2006	17071825
Purification and characterization of the three Snf1-activating kinases of Saccharomyces cerevisiae	Elbing et al.	2006	16201971
Subunits of the Snf1 kinase heterotrimer show interdependence for association and activity	Elbing et al.	2006	16847059
Transcriptional response of steady-state yeast cultures to transient perturbations in carbon source.	Ronen M, Botstein D.	2006	16381818
Integration of transcriptional and posttranslational regulation in a glucose signal transduction pathway in Saccharomyces cerevisiae	Kim et al.	2006	16400179
Regulatory domains of Snf1-activating kinases determine pathway specificity	Rubenstein et al.	2006	16607009
The transcriptional response of the yeast Na+-ATPase ENA1 gene to alkaline stress involves three main signaling pathways	Platara et al.	2006	17023428
Tpk3 and Snf1 protein kinases regulate Rgt1 association with Saccharomyces cerevisiae HXK2 promoter	Palomino et al.	2006	16528100
Nitrogen availability and TOR regulate the Snf1 protein kinase in Saccharomyces cerevisiae	Orlova et al.	2006	16980405
Tpk3 and Snf1 protein kinases regulate Rgt1 association with Saccharomyces cerevisiae HXK2 promoter	Palomino et al.	2006	16528100
Gis4, a new component of the ion homeostasis system in the yeast Saccharomyces cerevisiae	Ye et al.	2006	17030993
The regulation of autophagy in eukaryotic cells: do all roads pass through Atg1?	Stephan et al	2006	16874100
Snf1-dependent and Snf1-independent pathways of constitutive ADH2 expression in Saccharomyces cerevisiae	Voronkova et al.	2006	16415371
Differing responses of Gat1 and Gln3 phosphorylation and localization to rapamycin and methionine sulfoximine treatment in Saccharomyces cerevisiae	Kulkarni et al	2006	16487345
Snf1p-dependent Spt-Ada-Gcn5-acetyltransferase (SAGA) recruitment and chromatin remodeling activities on the HXT2 and HXT4 promoters	van Oevelen et al.	2006	16368692
AKINbetagamma contributes to SnRK1 heterotrimeric complexes and interacts with two proteins implicated in plant pathogen resistance through its KIS/GBD sequence	Gissot et al.	2006	17028154
Role of the iron mobilization and oxidative stress regulons in the genomic response of yeast to hydroxyurea	Dubacq et al	2006	16328372
Regulation of Snf1 in response to environmental stress	Hong et al.	2007	17438333
SIT4 regulation of Mig1p-mediated catabolite repression in Saccharomyces cerevisiae	Jin et al.	2007	18022394
Hxk2 regulates the phosphorylation state of Mig1 and therefore its nucleocytoplasmic distribution	Ahuatzi et al.	2007	17178716
Regulation of gluconeogenesis in Saccharomyces cerevisiae is mediated by activator and repressor functions of Rds2	Soontorngun et al.	2007	17875938
Regulation of snf1 protein kinase in response to environmental stress	Hong, Carlson	2007	17438333
Biochemical evidence for glucose-independent induction of HXT expression in Saccharomyces cerevisiae	Pasula et al.	2007	17586499
Crystal structure of the heterotrimer core of Saccharomyces cerevisiae AMPK homologue SNF1	Amodeo et al.	2007	17851534
Isolation of a novel complex of the SWI/SNF family from Schizosaccharomyces pombe and its effects on in vitro transcription in nucleosome arrays	Bernal et al.	2007	17508131
Rod1, an arrestin-related protein, is phosphorylated by Snf1-kinase in Saccharomyces cerevisiae	Shinoda et al.	2007	17949685
A proteomic screen reveals SCFGrr1 targets that regulate the glycolytic-gluconeogenic switch	Benati et al.	2007	17828247
Stress-responsive Gln3 localization in Saccharomyces cerevisiae is separable from and can overwhelm nitrogen source regulation	Tate et al	2007	17439949
Glucose-responsive regulators of gene expression in Saccharomyces cerevisiae function at the nuclear periphery via a reverse recruitment mechanism	Sarma et al	2007	17237508
The localization of nuclear exporters of the importin-beta family is regulated by Snf1 kinase, nutrient supply and stress	Quan et al.	2007	17544521
A poised initiation complex is activated by SNF1	Tachibana et al	2007	17974563
DNA sequences from Arabidopsis, which encode protein kinases and function as upstream regulators of Snf1 in yeast	Hey et al.	2007	17237223
A proteomic screen reveals SCFGrr1 targets that regulate the glycolytic-gluconeogenic switch	Benanti et al.	2007	17828247
N-myristoylation regulates the SnRK1 pathway in Arabidopsis	Pierre et al.	2007	17827350
The moss genes PpSKI1 and PpSKI2 encode nuclear SnRK1 interacting proteins with homologues in vascular plants	Thelander et al.	2007	17533513
The complement of protein kinases of the microsporidium Encephalitozoon cuniculi in relation to those of Saccharomyces cerevisiae and Schizosaccharomyces pombe	Miranda-Saavedra et al	2007	17784954
Roles of the glycogen-binding domain and Snf4 in glucose inhibition of SNF1 protein kinase	Momcilovic et al.	2008	18474591
The pathway by which the yeast protein kinase Snf1p controls acquisition of sodium tolerance is different from that mediating glucose regulation	Ye et al.	2008	18757815
Novel Ree1 regulates the expression of ENO1 via the Snf1 complex pathway in Sc.	Choi et al.	2008	18851946
Artificial recruitment of mediator by the DNA-binding domain of Adr1 overcomes glucose repression of ADH2 expression	Young et al.	2008	18250152
Nsf1/Ypl230w participates in transcriptional activation during non-fermentative growth and in response to salt stress in Saccharomyces cerevisiae	Hlynialuk et al.	2008	18667581
Access denied: Snf1 activation loop phosphorylation is controlled by availability of the phosphorylated threonine 210 to the PP1 phosphatase	Rubenstein et al.	2008	17991748
Physiological characterization of glucose repression in the strains with SNF1 and SNF4 genes deleted	Usaite et al.	2008	17949842
Time-dependent profiling of metabolites from Snf1 mutant and wild type yeast cells	Humston et al.	2008	18826242
Direct regulation of genes involved in glucose utilization by the calcium/calcineurin pathway	Ruiz et al.	2008	18362157
Genomewide screening for genes associated with gliotoxin resistance and sensitivity in Saccharomyces cerevisiae	Chamilos et al.	2008	18212113
Characterization of glucose transport mutants of Saccharomyces cerevisiae during a nutritional upshift reveals a correlation between metabolite levels and glycolytic flux	Bosch et al.	2008	18042231
Detection of endogenous Snf1 and its activation state: application to Saccharomyces and Candida species	Orlova et al.	2008	18949820
A chemical genomics study identifies Snf1 as a repressor of GCN4 translation	Shirra et al	2008	18955495
Cyclic AMP-protein kinase A and Snf1 signaling mechanisms underlie the superior potency of sucrose for induction of filamentation in Saccharomyces cerevisiae	Van de Velde et al.	2008	17890371
Promoter binding by the Adr1 transcriptional activator may be regulated by phosphorylation in the DNA-binding region	Kacherovsky et al.	2008	18791642
Characterization of global yeast quantitative proteome data generated from the wild-type and glucose repression saccharomyces cerevisiae strains: the comparison of two quantitative methods	Usaite et al.	2008	18173223
Cooperation of two mRNA-binding proteins drives metabolic adaptation to iron deficiency	Puig et al	2008	18522836
Cloning and expression analyses of sucrose non-fermenting-1-related kinase 1 (SnRK1b) gene during development of sorghum and maize endosperm and its	Jain et al	2008	18433416
Structural insight into the autoinhibition mechanism of AMP-activated protein kinase	Chen et al.	2009	19474788
Snf1 controls the activity of Adr1 through dephos of Ser230	Ratnakumar et al.	2009	19398770
The Snf1 kinase and proteasome-associated Rad23 regulate UV-responsive gene expression	Wade et al.	2009	19680226
The Hsp70 homolog Ssb is essential for glucose sensing via the SNF1 kinase network	von Plehwe et al.	2009	19723765
Reconstruction of the yeast Snf1 kinase regulatory network reveals its role as a global energy regulator	Usaite et al.	2009	19888214
A network biology approach to aging in yeast	Lorenz et al.	2009	19164565
Arabidopsis protein kinases GRIK1 and GRIK2 specifically activate SnRK1 by phosphorylating its activation loop	Shen et al.	2009	19339507
Methylglyoxal activates Gcn2 to phosphorylate eIF2alpha independently of the TOR pathway in Saccharomyces cerevisiae	Nomura et al.	2009	20077113
Mediator subunits and histone methyltransferase Set2 contribute to Ino2-dependent transcriptional activation of phospholipid biosynthesis in the yeast Saccharomyces cerevisiae	Dettman et al.	2009	20054697
Asymmetric signal transduction through paralogs that comprise a genetic switch for sugar sensing in Saccharomyces cerevisiae	Sabina et al.	2009	19720826
Trehalose Metabolites in Arabidopsis-elusive, active and central	Schluepmann et al.	2009	22303248
Genetic basis of arsenite and cadmium tolerance in Saccharomyces cerevisiae	Thorsen et al.	2009	19284616
Threonine at position 306 of the KAT1 potassium channel is essential for channel activity and is a target site for ABA-activated SnRK2/OST1/SnRK2.6 protein kinase	Sato et al.	2009	19785574
Mitochondrial function is an inducible determinant of osmotic stress adaptation in yeast	Pastor et al	2009	19720830
Inference of functional networks of condition-specific response--a case study of quiescence in yeast	Roy et al	2009	19209695
Snf1-independent, glucose-resistant transcription of Adr1-dependent genes in a mediator mutant of Saccharomyces cerevisiae	Young et al.	2009	19732343
Biochemical and functional studies on the regulation of the Sc SNF1	Amodeo et al.	2010	20529674
Roles of the Snf1-activating kinases during nitrogen limitation and pseudohyphal differentiation in Saccharomyces cerevisiae	Orlova et al.	2010	19880754
Glucose signaling-mediated coordination of cell growth and cell cycle in Saccharomyces cerevisiae	Busti et al.	2010	22219709
Functional domains of yeast Hxk2	Pelaez et al.	2010	20815814
The beta-subunits of the Snf1 kinase in Saccharomyces cerevisiae, Gal83 and Sip2, but not Sip1, are redundant in glucose derepression and regulation of sterol biosynthesis	Zhang et al.	2010	20545859
PP1 phosphatase binding motif in Reg1 protein of Sc. is required for interaction with both the PP1 phosphatase Glc7 and the Snf1 protein kinase	Tabba et al.	2010	20170726
Differential glucose repression in common yeast strains in response to HXK2 deletion	Kümmel et al.	2010	20199578
Differential glucose repression in common yeast strains in response to HXK2 deletion	Kümmel et al.	2010	20199578
Differential roles of the glycogen-binding domains of beta subunits in regulation of the Snf1 kinase complex	Mangat et al.	2010	19897735
An inhibited conformation for the protein kinase domain of the Saccharomyces cerevisiae AMPK homolog Snf1	Rudolph et al.	2010	20823513
Transcriptional regulation of nonfermentable carbon utilization in budding yeast.	Turcotte et al.	2010	19686338
Snf1 promotes phosphorylation of the alpha subunit of eukaryotic translation initiation factor 2 by activating Gcn2 and inhibiting phosphatases Glc7 and Sit4	Cherkasova et al.	2010	20404097
System biology of energy homeostasis in Yeast	Zhang et al.	2010	20439164
Isolation and characterization of the carbon catabolite-derepressing protein kinase Snf1 from the stress tolerant yeast Torulaspora delbrueckii	Hernández-López et al.	2010	20824888
The snf1 gene of Ustilago maydis acts as a dual regulator of cell wall degrading enzymes	Nadal et al.	2010	21062173
adaptation of yeast S.c and Brettanomyces bruxellensis to winemaking conditions: a comparative study of stress genes expression	Nardi et al.	2010	20730535
Synthesis and biological evaluation of combretastatin analogs as cell cycle	Coccetti et al.	2010	20363626
Snf1/AMPK promotes S-phase entrance by controlling CLB5 transcription in budding yeast	Pessina et al.	2010	20505334
Unraveling condition-dependent networks of transcription factors that control metabolic pathway activity in yeast	Fendt et al.	2010	21119627
14-3-3 (Bmh) proteins inhibit transcription activation by Adr1 through direct binding to its regulatory domain	Parua et al	2010	20855531
Regulatory mechanism for expression of GPX1 in response to glucose starvation and Ca in Saccharomyces cerevisiae: involvement of Snf1 and Ras/cAMP pathway in Ca signaling	Ohdate et al.	2010	20002498
Snf1p regulates Gcn5p transcriptional activity by antagonizing Spt3p	Liu et al.	2010	19841091
Growth signaling promotes chronological aging in budding yeast by inducing superoxide anions that inhibit quiescence	Weinberger et al	2010	21076178
Snf1 dependence of peroxisomal gene expression is mediated by Adr1	Ratnakumar et al.	2010	20139423
Roles of two protein phosphatases, Reg1-Glc7 and Sit4, and glycogen synthesis in regulation of SNF1 protein kinase	Ruiz et al.	2011	21464305
ADP regulates SNF1, the Sc homolog of AMPK	Mayer et al.	2011	22019086
Acetylation of yeast AMPK controls intrinsic aging independently of caloric restriction	Lu et al.	2011	21906795
Reg1 Protein Regulates Phosphorylation of All Three Snf1 Isoforms but Preferentially Associates with the Gal83 Isoform	Zhang et al.	2011	22002657
Interaction of SNF1 protein kinase with its activating kinase Sak1	Liu et al.	2011	21216941
Subunit and domain requirements for adenylate-mediated protection of Snf1 kinase activation loop from dephosphorylation	Chandrashekarappa et al.	2011	22065577
Galactose induction of the GAL1 gene requires conditional degradation of the Mig2 repressor	Lim et al.	2011	21323640
Alterations at dispersed sites cause phosphorylation and activation of SNF1 protein kinase during growth on high glucose	Momcilovic et al.	2011	21561858
Shifting the fermentative/oxidative balance in Saccharomyces cerevisiae by transcriptional deregulation of Snf1 via overexpression of the upstream activating kinase Sak1p	Raab et al.	2011	21257817
Subunit and domain requirements for adenylate mediated protection of Snf1 kinase activation loop from dephosphorylation	Chandrashekarappa et al.	2011	22065577
Ubp8 and SAGA regulate Snf1 AMP kinase activity	Wilson et al.	2011	21628526
Structure of mammalian ANPK and its regulation by ADP	Xiao et al.	2011	21399626
ADP regulates SNF1, the Saccharomyces cerevisiae homolog of AMP-activated protein kinase.	Mayer et al.	2011	22019086
Cell biology. Why starving cells eat themselves	Hardie et al.	2011	21273476
Toward a global analysis of metabolites in regulatory mutants of yeast	Humston et al.	2011	21416166
Activator-independent transcription of Snf1-dependent genes in mutants lacking histone tails	Infante et al.	2011	21338416
A heuristic model for paradoxical effects of biotin starvation on carbon metabolism genes in the presence of abundant glucose	Velazquez-Arellano et al.	2011	20869286
Mapping the interaction of Snf1 with TORC1 in Saccharomyces cerevisiae	Zhang et al.	2011	22068328
Phenomic and transcriptomic analyses reveal that autophagy plays a major role in desiccation tolerance in Saccharomyces cerevisiae	Ratnakumar et al.	2011	20963216
The cAMP-dependent protein kinase signaling pathway is a key regulator of P body foci formation	Ramachandran et al.	2011	21925385
GABA induction of the Saccharomyces cerevisiae UGA4 gene depends on the quality of the carbon source: Role of the key transcription factors acting in this process	Levi et al.	2012	22525679
Protein Kinase A Contributes to the Negative Control of Snf1 protein kinase in Saccharomyces cerevisiae	Barrett et al.	2012	22140226
The role of the Snf1 kinase in the adaptive response of Saccharomyces cerevisiae to alkaline pH stress	Casamayor et al.	2012	22372618
Heterotrimer-independent regulation of activation-loop phosphorylation of Snf1 protein kinase involves two protein phosphatases	Ruiz et al.	2012	22589305
The regulation of filamentous growth in yeast	Cullen et al.	2012	22219507
Regulation of conditional gene expression by coupled transcription repression and RNA degradation	Lavoie et al.	2012	21933814

A molecular switch on an arrestin-like protein relays glucose signaling to transporter endocytosis	Becuwe et al.	2012	22249293
Snf1/AMPK regulates Gcn5 occupancy, H3 acetylation and chromatin remodelling at S. cerevisiae ADY2 promoter	Abate et al.	2012	22306658
Heterotrimer-independent regulation of activation-loop phosphorylation of Snf1 protein kinase involves two protein phosphatases.	Ruiz et al.	2012	22589305
Nuclear import of the yeast hexokinase 2 protein requires α/β-importin-dependent pathway.	Peláez et al.	2012	22157003
Metabolic activation of the HOG MAP kinase pathway by Snf1/AMPK regulates lipid signaling at the Golgi.	Piao et al.	2012	22882253
Phosphorylation of yeast hexokinase 2 regulates its nucleocytoplasmic shuttling.	Fernández-García et al.	2012	23066030
Glucose-induced posttranslational activation of protein phosphatases PP2A and PP1 in yeast.	Castermans et al	2012	22290422
The filamentous growth MAPK Pathway Responds to Glucose Starvation Through the Mig1/2 transcriptional repressors in Saccharomyces cerevisiae.	Karunanithi et al.	2012	22904036
Protein kinase A contributes to the negative control of Snf1 protein kinase in Saccharomyces cerevisiae	Barrett et al	2012	22140226
The AMP-activated protein kinase Snf1 regulates transcription factor binding, RNA polymerase II activity, and mRNA stability of glucose-repressed genes in Saccharomyces cerevisiae.	Young et al.	2012	22761425
Genome-wide location analysis reveals an important overlap between the targets of the yeast transcriptional regulators Rds2 and Adr1.	Soontorngun et al.	2012	22687600
The role of the Snf1 kinase in the adaptive response of Saccharomyces cerevisiae to alkaline pH stress	Casamayor et al.	2012	22372618
The regulation of filamentous growth in yeast.	Cullen et al.	2012	22219507
An Overview of Autophagy and Yeast Pseudohyphal Growth: Integration of Signaling Pathways during Nitrogen Stress	Song et al	2012	24710476
Reciprocal phosphorylation of yeast glycerol-3-phosphate dehydrogenases in adaptation to distinct types of stress.	Lee et al	2012	22988299
Nutritional control of growth and development in yeast.	Broach	2012	22964838
PAS kinase: integrating nutrient sensing with nutrient partitioning.	Cardon et al	2012	22245833
The core regulation module of stress-responsive regulatory networks in yeast.	Kim et al.	2012	22784859
Snf1-like protein kinase Ssp2 regulates glucose derepression in Schizosaccharomyces pombe.	Matsuzawa et al.	2012	22140232
N-terminal domain of nuclear IL-1α shows structural similarity to the C-terminal domain of Snf1 and binds to the HAT/core module of the SAGA complex.	Zamostna et al.	2012	22879895
Regulation of yeast pyruvate kinase by ultrasensitive allostery independent of phosphorylation.	Xu et al	2012	22902555
Integrated analysis of transcriptome and lipid profiling reveals the co-influences of inositol-choline and Snf1 in controlling lipid biosynthesis in yeast.	Chumnanpuen et al.	2012	22622761
Ligand binding to the AMP-activated protein kinase active site mediates protection of the activation loop from dephosphorylation.	Chandrashekarappa et al.	2013	23184934
Protein kinase Snf1 / AMPK : a new regulator of G1 / S transition in Saccharomyces cerevisiae	Degli et al.	2013 -	
The AMPK Family Member Snf1 Protects Saccharomyces cerevisiae Cells upon Glutathione Oxidation	Pérez-Sampietro et al	2013	23472170
Alteration of plasma membrane organization by an anticancer lysophosphatidylcholine analogue induces intracellular acidification and internalization of plasma membrane transporters in yeast.	Czyz et al.	2013	23344949
The FOX transcription factor Hcm1 regulates oxidative metabolism in response to early nutrient limitation in yeast. Role of Snf1 and Tor1/Sch9 kinases.	Rodríguez-Colman et al.	2013	23481038
Differential regulation of glucose transport activity in yeast by specific cAMP signatures.	Bermejo et al.	2013	23495665
Spatial reorganization of yeast enolase to alter carbon metabolism under hypoxia.	Miura et al.	2013	23748432
SUMOylation regulates the Snf1 protein kinase	Simpson-Lavy et al.	2013	24108357
Ptc1 protein phosphatase 2C contributes to glucose regulation of SNF1/AMP-activated protein kinase (AMPK) in Saccharomyces cerevisiae.	Ruiz et al.	2013	24019512
Protein Kinase A is part of a mechanism that regulates nuclear re-import of the nuclear tRNA export receptors Los1p and Msn5p.	Pierce JB, van der Merwe G, Mangroo D.	2013	24297441
The AMPK Family Member Snf1 Protects Saccharomyces cerevisiae Cells upon Glutathione Oxidation.	Pérez-Sampietro M, Casas C, Herrero E.	2013	23472170
Nucleotide degradation and ribose salvage in yeast.	Xu et al	2013	23670538
Reducing Sphingolipid Synthesis Orchestrates Global Changesto Extend Yeast Lifespan.	Liu et al	2013	23725375
Spatial reorganization of yeast enolase to alter carbon metabolism under hypoxia.	Miura et al.	2013	23748432
Assessment of crosstalks between the Snf1 kinase complex and sphingolipid metabolism in S. cerevisiae via systems biology approaches.	Borklu et al	2013	24056632
Snf1/AMPK promotes SBF and MBF-dependent transcription in budding yeast.	Busnelli et al.	2013	24084603
The yeast AMPK homolog SNF1 regulates acetyl-CoA homeostasis and histone acetylation.	Zhang et al.	2013	24081331
Reducing signs of aging and increasing lifespan by drug synergy.	Huang et al.	2013	23601176
The Impacts of Medium Acidity on Chronological Life Span of Saccharomyces cerevisiae: Lipids, Signaling Cascades, Mitochondrial and Vacuolar Functions.	Yucel et al.	2013	24056632
A Saccharomyces cerevisiae assay system to investigate ligand/AdipoR1 interactions that lead to cellular signaling.	Aouida et al.	2013	23762377
Crosstalk between SNF1 Pathway and the Peroxisome-Mediated Lipid Metabolism in Magnaporthe oryzae.	Zeng et al.	2013	25090011
Regulation of Yeast G Protein Signaling by the Kinases That Activate the AMPK Homolog Snf1	Clement, et al.	2013	24003255
Mutations in SNF1 complex genes affect yeast cell wall strength.	Backhaus et al.	2014	24486034
Nutrient sensing and signaling in the yeast Saccharomyces cerevisiae.	Conrad et al.	2014	24483210
Glucose derepression by yeast AMP-activated protein kinase SNF1 is controlled via at least two independent steps.	Garcia-Salcedo et al.	2014	24529170
Yeast AMP-Activated Protein Kinase Monitors Glucose Concentration Changes as well as Absolute Glucose Levels.	Bendrioua et al.	2014	24627493
Rewiring AMPK and Mitochondrial Retrograde Signaling for Metabolic Control of Aging and Histone Acetylation in Respiratory-Defective Cells.	Frijs et al.	2014	24726357
Improving production of malonyl coenzyme a-derived metabolites by abolishing snf1-dependent regulation of acc1.	Shi et al.	2014	24803522
The mammalian AMP-activated protein kinase complex mediates glucose regulation of gene expression in the yeast Saccharomyces cerevisiae.	Tian et al.	2014	24815694
A dedicated database system for handling multi-level data in systems biology.	Pornputtapong et al.	2014	25053973
Improving Polyketide and Fatty Acid Synthesis by Engineering of the Yeast Acetyl-CoA Carboxylase.	Choi et al.	2014	25078432
State Transitions in the TORC1 Signaling Pathway and Information Processing in Saccharomyces cerevisiae.	Hughes et al.	2014	25085507
Genetic Analysis of Resistance and Sensitivity to 2-deoxyglucose in Saccharomyces cerevisiae.	McCartney et al.	2014	25116136
Regulation of gene expression through a transcriptional repressor that senses acyl-chain length in membrane phospholipids.	Hofbauer et al.	2014	24960695
Yeast cytotoxic sensitivity to the antitumour agent β-lapachone depends mainly on oxidative stress and is largely independent of microtubule- or topoisomerase-mediated DNA damage.	Ramos-Pérez et al.	2014	24726357
Co-regulated expression of Na+/phosphate Pho89 transporter and Ena1 Na+-ATPase allows their functional coupling under high pH stress.	Serra-Cardona et al.	2014	25266663
14-3-3 functions as a comodulator of transcription by inhibiting coactivator functions.	Parua et al	2014	25355315
PAS kinase is activated by direct Snf1-dependent phosphorylation and mediates inhibition of TORC1 through the phosphorylation and activation of Pbp1.	DeMille et al.	2014	25428989
Fine-Tuning of Histone H3 Lys4 Methylation During Pseudohyphal Differentiation by the CDK Submodule of RNA Polymerase II.	Law et al.	2014	25467068
Pleiotropy of the de novo-originated gene MDF1.	Li et al.	2014	25452167
Leveraging transcription factors to speed cellobiose fermentation by <i>Saccharomyces cerevisiae.</i>	Lin et al.	2014	25435910
2-Deoxyglucose impairs yeast growth by stimulating Snf1-regulated and α-arrestin-mediated trafficking of hexose transporters 1 and 3 in Saccharomyces cerevisiae.	O'Donnell et al.	2014	25547292
Protein kinase Ymr291w/Tda1 is essential for glucose signaling in Saccharomyces cerevisiae on the level of hexokinase isoenzyme ScHxk2 phosphorylation.	Kaps et al.	2015	25593311
